# Spatial Association Between Polyamine Metabolism and Lipid Peroxidation in Different Pathological Regions of Glioblastoma

**DOI:** 10.3390/biomedicines14092067

**Published:** 2026-09-15

**Authors:** Zheng Qu, Kaijing Guo, Xin Xiang, Yuhan An, Ting Lei, Angsi Liu, Yulin Wang, Dongting Chen, Hong Chen, Ziqian Zhang, Jiangjia Qin, Jiuming He, Xueji Li, Fangjun Liu, Yan Li

**Affiliations:** 1 Beijing Key Laboratory of New Drug Mechanisms and Pharmacological Evaluation Study, Department of Pharmacology, Institute of Materia Medica, Chinese Academy of Medical Sciences & Peking Union Medical College, Beijing 100050, China; 2 State Key Laboratory of Bioactive Substance and Function of Natural Medicines, Institute of Materia Medica, Chinese Academy of Medical Sciences & Peking Union Medical College, Beijing 100050, Chinahejiuming@imm.ac.cn (J.H.); 3 Department of Neurosurgery, Sanbo Brain Hospital, Capital Medical University, Beijing 100018, China; 4 Department of Neurosurgery, National Cancer Center/National Clinical Research Center for Cancer/Cancer Hospital, Chinese Academy of Medical Sciences & Peking Union Medical College, Beijing 100021, Chinali-x-j@163.com (X.L.)

**Keywords:** glioblastoma, polyamine metabolism, ferroptosis, spatial metabolomics

## Abstract

**Background**: Glioblastoma is highly heterogeneous, highly invasive, and resistant to temozolomide, with an extremely low 5-year survival rate. The spatial association between polyamine metabolism and ferroptosis remains unclear. Spatial metabolic heterogeneity is a core challenge in glioblastoma treatment. **Methods**: In this study, 17 patients with primary or recurrent high-grade glioma were included. Spatial metabolomics (*n* = 9), multiplex immunofluorescence (exploratory experiment *n* = 10, observational experiment *n* = 1), flow cytometry (*n* = 13), and mouse orthotopic models (*n* = 3) were combined to analyze the characteristics of the polyamine–ferroptosis axis in the para-carcinoma, tumor, and necrotic regions. **Results**: Differences in the polyamine-metabolism–ferroptosis axis were observed across distinct pathological regions of glioblastoma. Regional differences in polyamine distribution were associated with regional differences that lower lipid-peroxidation signals in the tumor region and higher lipid-peroxidation signals in the necrotic region. Moreover, in glioblastoma patient specimens, lipid-peroxidation-associated signals were detected in immune-cell compartments, alongside immune-cell phenotypic features suggestive of dysfunction, which show correlation with an immunosuppressive microenvironment. At the regional level, enzymes related to polyamine metabolism, namely GLS and SMS, exhibited spatial co-localization trends with lipid-peroxidation markers. Alterations in markers of the GSH-GPX4 and FSP1 antioxidant systems were spatially associated with the tumor-region-specific lipid-peroxidation profile. Partial spatial patterns of polyamine metabolism observed in human samples were partially recapitulated in mouse orthotopic tumor models. **Conclusions**: Glioblastoma exhibits spatially distinct distribution of the polyamine–ferroptosis-associated molecular signature. Notable correlative spatial features include spermidine and spermine enrichment, altered regional abundance within the glutamine–glutamate metabolic axis, immune-cell lipid-peroxidation-associated signals and immune-cell-dysfunction-related phenotypes, and regional shifts in GSH-GPX4/FSP1-related markers. This study identifies potential targets and provides a correlative spatial framework for region-specific therapeutic investigation, but the establishment of the causal relationship still requires further functional verification.

## 1. Introduction

Glioblastoma (GBM) is the most common primary malignant brain tumor, characterized by extreme heterogeneity, high invasiveness, and resistance to temozolomide (TMZ), and the 5-year survival rate remains below 10% [1,2,3,4,5]. Intratumoral heterogeneity at genetic, transcriptomic and functional levels is a core challenge, limiting the efficacy of single therapies [6,7].

In recent years, ferroptosis, an iron-dependent form of regulated cell death driven by lipid peroxidation, is tightly linked to GBM drug resistance and recurrence. It is controlled by antioxidant systems (e.g., the GPX4/xCT axis) and metabolites including glutathione and polyamines [8,9]. Tumors like GBM often upregulate antioxidant defenses to resist ferroptosis, supporting proliferation, coping with oxidative stress, and driving therapeutic resistance [10,11].

Polyamine metabolism (involving spermidine and spermine) has a dual role in ferroptosis: polyamines themselves exert antioxidant effects, but their acetylation promotes reactive oxygen species (ROS) and lipid peroxidation, positively regulating ferroptosis. This duality suggests tumor cells may spatially reprogram polyamine flux to fine-tune ferroptosis sensitivity across regions for survival [12,13]. Furthermore, ferroptosis is closely involved in the shaping of the tumor immune microenvironment and may affect the infiltration and function of immune cells [14]. However, at present, systematic research on the polyamine–ferroptosis axis in GBM, especially spatial dynamics and mechanisms, is lacking.

Spatial metabolomics enables direct metabolite distribution analysis in situ while preserving tissue architecture, providing a powerful tool to resolve GBM metabolic heterogeneity and link metabolism to cell fate [15]. This study integrated spatial metabolomics, multiplex immunofluorescence, flow cytometry, and animal models to map polyamine–ferroptosis axis characteristics across para-carcinoma (P), tumor (T), and geographic-dominant necrosis (B) regions of GBM. We depicted the spatial association between polyamine metabolism and ferroptosis in different pathological regions of GBM. These findings not only deepen the understanding of the metabolic heterogeneity of GBM but also provide potential targets for region-specific therapy targeting the polyamine-metabolism–ferroptosis axis, especially in TMZ-resistant GBM.

## 2. Materials and Methods

### 2.1. Study Sample

This study included 17 enrolled patients forming a high-grade glioma cohort who underwent surgery at Sanbo Brain Hospital, Capital Medical University, Beijing, China, and received pathological confirmation of GBM. Among them, 11 patients had a primary diagnosis of GBM, and 6 patients had a recurrence of GBM. Informed consent was obtained from all participants or their parents or legal guardians. This study was approved by the Institutional Review Board of Sanbo Brain Hospital (Approval Nos.: SBNK-YJ-2024-029-02, SBNK-YJ-2025-003-01). All methods were conducted in accordance with the guidelines of the Institutional Review Board of Sanbo Brain Hospital and the Declaration of Helsinki. All patient information can be found in Table S1. IDH mutation status for participants is summarized in Table S1. IDH-mutant glioma specimens were excluded from the spatial metabolomics analysis due to their distinct tumor metabolic characteristics. Complete pre-surgical treatment history and MGMT promoter-methylation results could not be retrospectively retrieved for this archival cohort.

### 2.2. H&E Staining

The tissue sections were incubated at 65 °C for 2 h, deparaffinized with xylene, and rehydrated with an ethanol gradient (100–50%). Next, the sections were stained with hematoxylin solution for 5 min, dipped in 1% acid ethanol (1% HCl in 70% ethanol), and then rinsed with distilled water. Afterward, the sections were stained with an eosin solution for 3 min, dehydrated with ethanol, and cleared in xylene.

### 2.3. Tissue Regional Definition

Three histological regions, including the P, T, and B regions, were strictly defined based on H&E staining and professional neuropathological evaluation. The P region was defined as the tumor-adjacent brain tissue zone outside the bulk tumor mass, which typically contains infiltrating tumor cells, reactive astrocytes, activated microglia, vasogenic edema, and abnormal vascular remodeling, rather than normal brain tissue. The T region was characterized by densely distributed atypical tumor cells, obvious nuclear pleomorphism, and active proliferation without large-area necrosis. The B region was defined as an acellular zone with extensive karyolysis, tissue fragmentation, and typical pseudopalisading necrosis. All regional annotations were independently performed by a senior neuropathologist who was fully blinded to the MSI data and immunofluorescence results to avoid assessment bias. In the present study, the B regions are predominantly geographic necrosis. Small pseudopalisading necrotic micro-foci were sporadically observed in some sections but were not segmented as independent regions of interest for statistical analysis, due to their limited spatial dimension on frozen tissue sections. Spatial matching among serial tissue sections was achieved by registering tissue contours, morphological features, vascular distribution, and necrotic lesion topography. This strategy ensured consistent spatial correspondence among H&E staining, MSI acquisition, and immunofluorescence analysis across adjacent sections.

### 2.4. Spatial Metabolomics Detection of Tumor Tissues in Mouse Models and Patients with GBM

The brain tissues of the parent murine GL261 group, tumors derived from the in vivo TMZ-selected GL261 line orthotropic models, and the GBM tumor tissues derived from patients used for spatial metabolism detection were frozen at −80 °C. When sectioning, the tissue was removed from the ultralow temperature refrigerator at −80 °C and thawed at −20 °C for 2 h, after which cross-sectional sections (12 μm thick) were attached to positively charged anti-disengagement slides. Tissue sections were vacuum-dried and mounted onto the sample stage. Mass spectrometry imaging and data acquisition were performed using the AFADESI-MSI (Air-flow-Assisted Desorption Electrospray Ionization-Mass Spectrometry Imaging) platform.

MSI analysis was performed in positive and negative ion mode on a home-built AFADESI platform consisting of the AFADESI ion source and a Q-Orbitrap mass spectrometer (Q-Exactive, Thermo Fisher Scientific, Waltham, MA, USA). AFADESI is an ambient open-air desorption/ionization source operating under atmospheric-pressure conditions. MSI data were acquired in both positive ion and negative ion modes. The mass-to-charge acquisition range was set to *m*/*z* 70–1000. The Orbitrap mass analyzer was operated at a mass resolution of 70,000. The spatial resolution for tissue imaging was set to 100 μm for both human GBM frozen sections and mouse brain frozen sections. Mass calibration was performed prior to data acquisition using the instrument-provided external calibration mix. All human glioblastoma tissue sections were measured within a single experimental acquisition batch. During acquisition, consecutive scanning lines were collected across the tissue surface, and each scan line was saved as an individual raw data file.

### 2.5. Bioinformatics Analysis

The overall metabolic network was constructed using overall kinetic matter spectroscopy imaging data. First, the mass spectrometry data of each tissue sample were extracted using MassImager software (MassImager Pro v1.0). Raw MSI data were processed using MarkView software (AB SCIEX, Farmingham, MA, USA). Normalization was performed using the total ion current (TIC): each mass spectrum was divided by its total sum of intensities. No endogenous metabolite was used as a normalization reference. In this GBM dataset, tissue sections consist of three highly divergent pathological compartments: the P, T and B regions. Candidate house-keeping endogenous metabolites display substantial spatial-abundance fluctuations across these heterogeneous microregions. No endogenous metabolite maintains uniformly stable intensity across the P, T and B regions, so endogenous-metabolite-based normalization would introduce systematic bias and misrepresent true regional metabolic alterations. Consequently, TIC normalization was selected for the present study. We note that TIC normalization cannot completely eliminate pixel-to-pixel variation in ionization efficiency, which represents an inherent limitation of ambient AFADESI-MSI measurement. Following peak alignment and normalization in MarkView software (MarkerView^TM^ v1.3.1), an *m*/*z* and peak intensity data matrix was generated. *m*/*z* data from positive and negative ion modes were imported into MetaboAnalyst 6.0 (https://www.metaboanalyst.ca/, accessed on 1 September 2025). The volume difference was set to 5 PPM, and charge-to-mass mixed ion mode was used. After screening the additional ion forms set within the website, high-throughput matching was conducted on the original data to determine the final list of putatively annotated metabolites, obtain the pathway information of significant changes, and construct and visualize the metabolic network of the obtained metabolites in combination with the KEGG database and Cytoscape 3.7.1. Key metabolites including spermine, spermidine, putrescine, glutamine and glutamate represent putatively annotated metabolites, assigned by accurate-monoisotopic-mass matching (5 ppm mass tolerance) and isotope-pattern consistency evaluation according to MSI reporting conventions. On tissue MS/MS, fragmentation and authentic-standard validation were not performed on these GBM tissue sections. While these metabolites have been independently validated by LC-MS/MS with authentic standards in other human tissues from our group, such external orthogonal evidence cannot definitively confirm compound identity within individual GBM pixels. Confidence intervals for individual metabolite assignments are not available in the present dataset.

### 2.6. Flow Cytometry

The protective solution was removed from the tumor tissue using 1640 medium. The tissue was digested with tissue lysis buffer. After centrifugation and discarding the supernatant, the precipitate was washed with medium and filtered through a 70-micron filter. Red blood cell lysis buffer was added to lyse the red blood cells, which were then incubated at room temperature for 20 min. 1 × 10^6^ cells were added to the antibody-conjugated cell membrane staining solution and incubated for 30 min. Afterward, the cells were washed with the lysis agent, and intracellular staining was conducted in the dark for another 30 min. The antibodies used were as follows: PerCP eFluor 710-CD3 (46-0037-41; Invitrogen, Thermo Fisher Scientific, Waltham, MA, USA), FITC-CD4 (11-0048-42; Invitrogen), Alexa Fluor 532-CD8a (58-0088-42; Invitrogen), and PE Cy7-CD279 (329917; Biolegend, San Diego, CA, USA). After passing through a 40-micron cell filter, machine detection was performed (NL-CLC B14, CYTEK, Fremont, CA, USA). Gating strategy: First, a forward-scatter area (FSC-A) versus side-scatter area (SSC-A) dot plot was used to identify the total cell population and exclude debris and small-particle noise. Next, FSC-H versus FSC-A was applied to gate single cells by removing cell doublets and multiplet aggregates. Within the single-cell population, viability dye signals were used to exclude dead or apoptotic cells, yielding the population of total viable single cells. Subsequently, immune-cell subsets were further gated from total viable single cells according to the expression pattern of cell-surface markers. All target immune populations were identified sequentially following this hierarchical gating workflow. Dead cells were excluded using a commercial fixable viability dye. Events positive for viability dye were regarded as non-viable and discarded from downstream analysis. Single-stained compensation control tubes were prepared for each fluorescent channel, including unstained controls, single antibody-stained controls, and viability-dye-only controls. A compensation matrix was calculated and applied before sample acquisition to correct fluorescence spectral overlap across different channels. Only measurements derived from ≥100 events within the gated target immune subset (the denominator used for percentage calculation) were considered reliable and included in downstream statistical comparisons. This threshold does not refer to total acquired cellular events. Values calculated from fewer than 100 events in the gated target subset were flagged and excluded from statistical analysis. The abundance of each immune-cell subset was calculated as a percentage relative to total viable single cells. No normalization by tissue mass was performed in this study. All flow cytometry data were analyzed using FlowJo software (FlowJo v10.8.1).

### 2.7. Multiplex Immunofluorescence Staining (TSA Staining)

The FFPE sections were incubated at 65 °C for 2 h and then dewaxed and hydrated with xylene and gradient alcohol. EDTA buffer (pH 9.0) or sodium citrate buffer (pH 6.0) was used for antigen retrieval. For each primary antibody, the optimized antigen-retrieval conditions are as follows: anti-FSP1 (EDTA buffer, pH 9.0, microwave boiling for 15 min); anti-GLS (sodium citrate buffer, pH 6.0, microwave boiling for 15 min); anti-GPX4 (EDTA buffer, pH 9.0, microwave boiling for 20 min); anti-SMS (sodium citrate buffer, pH 6.0, microwave boiling for 15 min); anti-Iba1 (EDTA buffer, pH 9.0, microwave boiling for 20 min); anti-4-HNE (sodium citrate buffer, pH 6.0, microwave boiling for 15 min); and anti-GFAP (EDTA buffer, pH 9.0, microwave boiling for 15 min). The sections were preincubated with 10% goat serum to block nonspecific binding sites. A multi-IHC staining kit (Baiqiandu Biotechnology, Wuhan, China) was used according to the manufacturer’s instructions. The slides were incubated first with the primary antibody and then with the secondary antibody, and tyrosine signal amplification was performed. The sections were subsequently subjected to heat repair in a microwave, after which the next round of staining was performed. After all the indicators were stained, DAPI was used to mark the nucleus. Pannoramic SCAN and 3DHISTECH CaseViewer (3DHISTECH, Budapest, Hungary) were used for multispectral slice scanning and image analysis. The antibodies used were as follows: anti-FSP1 (A22278, ABclonal, Wuhan, China), anti-GLS (MA5-54013, Thermo Fisher, Waltham, MA, USA), anti-GPX4 (ab125006, Abcam, Cambridge, UK), anti-SMS (ab151547, Abcam, Cambridge, UK), anti-Iba1 (17198T, Cell Signaling Technology, Danvers, MA, USA), anti-4-HNE (ab48506, Abcam, Cambridge, UK), and anti-GFAP (ab278054, Abcam, Cambridge, UK).

### 2.8. Preparation and Validation of Tumors Derived from the In Vivo TMZ-Selected GL261 Line Mouse Model

This experiment was carried out after being approved by The Animal Care & Welfare Committee of the Institute of Materia Medica, CAMS & PUMC. Through continuous and multigeneration treatment with TMZ in vivo, resistance to TMZ was induced in mouse GL261 glioblastoma. Drug resistance was subsequently evaluated using an ectopic transplanted tumor model (Animal Qualification Certificate Number: 111251211100077643, Animal Experiment Ethics Review Form Number: 00009020.) This experiment included seven mice in each group. The results revealed that after continuous administration of 50.0 mg/kg TMZ for five days, the sensitivity of GL261 tumors in the drug-resistant model group mice to TMZ was significantly lower than that in the parental group. This verified the successful establishment of the GL261 glioblastoma model in tumors derived from the in vivo TMZ-selected GL261 line mice and provided an in vivo model of TMZ resistance for subsequent experiments. It should be emphasized that the evidence from subcutaneous tumor observation is limited. In this study, orthotopic tumors derived from these cells are solely used to explore spatial metabolic features, and are not used for assessing in vivo temozolomide therapeutic efficacy. Formal characterizations including in vitro IC_50_, resistance index, phenotype stability assessment, molecular dissection of resistance mechanisms, and direct proof of a maintained drug-resistance phenotype after intracranial orthotopic implantation have not been performed in the current work.

### 2.9. Preparation of Parent and Tumor Tissues Derived from the In Vivo TMZ-Selected GL261 Line Murine Orthotropic Models

This experiment was carried out after being approved by The Animal Care & Welfare Committee of the Institute of Materia Medica, CAMS & PUMC. Male C57BL/6J mice were selected (Animal Qualification Certificate Number: 110322231103824417; Animal Experiment Ethics Review Form Number: 00009172). This experiment included three mice in each group. Tumor tissues harvested from subcutaneously passaged parental and tumor groups derived from the in vivo TMZ-selected GL261 line-bearing mice were processed inside a laminar flow biosafety cabinet. Afterward, they were ground using a glass homogenizer, centrifuged at 1500 rpm for 5 min, resuspended and washed three times with sterile physiological saline. The cells in the suspension were counted under a microscope. The concentrations of the tumor suspensions in the parental group and the tumors derived from the in vivo TMZ-selected GL261 line group were adjusted to be the same using sterile normal saline and kept on ice for later use. The mice were anesthetized by intraperitoneal injection of pentobarbital sodium at a dosage of 50.0 mg/kg. After the hair at the top of the skull was shaved, the skin in the surgical area was disinfected with alcohol-soaked cotton balls and then fixed to the stereotactic brain. After an incision approximately 5 mm long was made with a scalpel, the incision was cleaned with a sterile medical cotton swab dipped in 10% hydrogen peroxide, the anterior fontanelle point of the skull was exposed, and a hole was drilled precisely. A total of 5 × 10^5^ GL261 glioma cells in 1 μL sterile PBS were stereotaxically injected into the right striatum with the following coordinates relative to the bregma: anteroposterior +0.5 mm, mediolateral +2.0 mm, dorsoventral −3.0 mm. Mice were maintained for 14 days after tumor cell implantation. All animals were sacrificed at the pre-defined 14-day time-point for brain tissue harvesting and subsequent spatial metabolomics and histological analysis.

All animal experiments described in Section 2.7 and Section 2.8 were approved by the Institutional Animal Care and Use Committee. Mice were randomly allocated into experimental groups using random number generation before tumor implantation. Investigators were blinded during tumor-bearing animal screening and tissue outcome evaluation. Animals that suffered severe weight loss (>20% body weight), premature death before planned endpoint, or failed tumor engraftment upon post-mortem pathological examination were excluded from further analysis (pre-defined inclusion–exclusion criteria). Mice were monitored daily after intracranial tumor implantation for body weight loss, activity level, neurological deficits (including limb weakness, ataxia, and impaired feeding), and overall physical condition. Pre-defined humane endpoints included sustained body weight loss exceeding 20% of initial body weight, obvious neurological dysfunction, persistent reduced food intake, and marked deterioration of general status. Once animals reached any humane-endpoint criterion, they were sacrificed immediately. Euthanasia was performed via carbon-dioxide inhalation followed by cervical dislocation to confirm death.

### 2.10. Statistical Processing

The experimental results are expressed as the mean ± standard deviation, and data analysis was performed using GraphPad Prism (8.4.0) software. Before statistical testing, normality of data distribution was assessed via Shapiro–Wilk test, and homogeneity of variance was evaluated using Levene’s test. For paired comparisons of repeated measurements obtained from the same subject, mixed-effects models with patient or mouse as random effects were applied accordingly. Model formula: Metabolite_intensity~Region + (1|PatientID). Fixed-effect term: Region (three levels: P, T, B). Random-effect term: random intercept for “PatientID”, accounting for within-patient correlation of repeated regional technical measurements. Hierarchical observations: nine independent biological patients; multiple nested pixel-level technical observations within each patient. Degrees of freedom were calculated using the Satterthwaite approximation implemented within the lmerTest package. Statistical inference was performed at the patient level, treating the patient as the biological unit, while pixels served only as nested technical replicates. For multi-group comparisons among independent samples satisfying parametric assumptions, one-way ANOVA followed by Tukey’s post hoc test was performed. Observations derived from single-specimen illustrative multiplex immunofluorescence images are descriptive only, and no statistical inference was performed. A value of *p* < 0.05 was considered statistically significant.

## 3. Results

### 3.1. Identification of Spatial Metabolic Characteristics in Different Pathological Microregions of GBM

**Figure 1:**
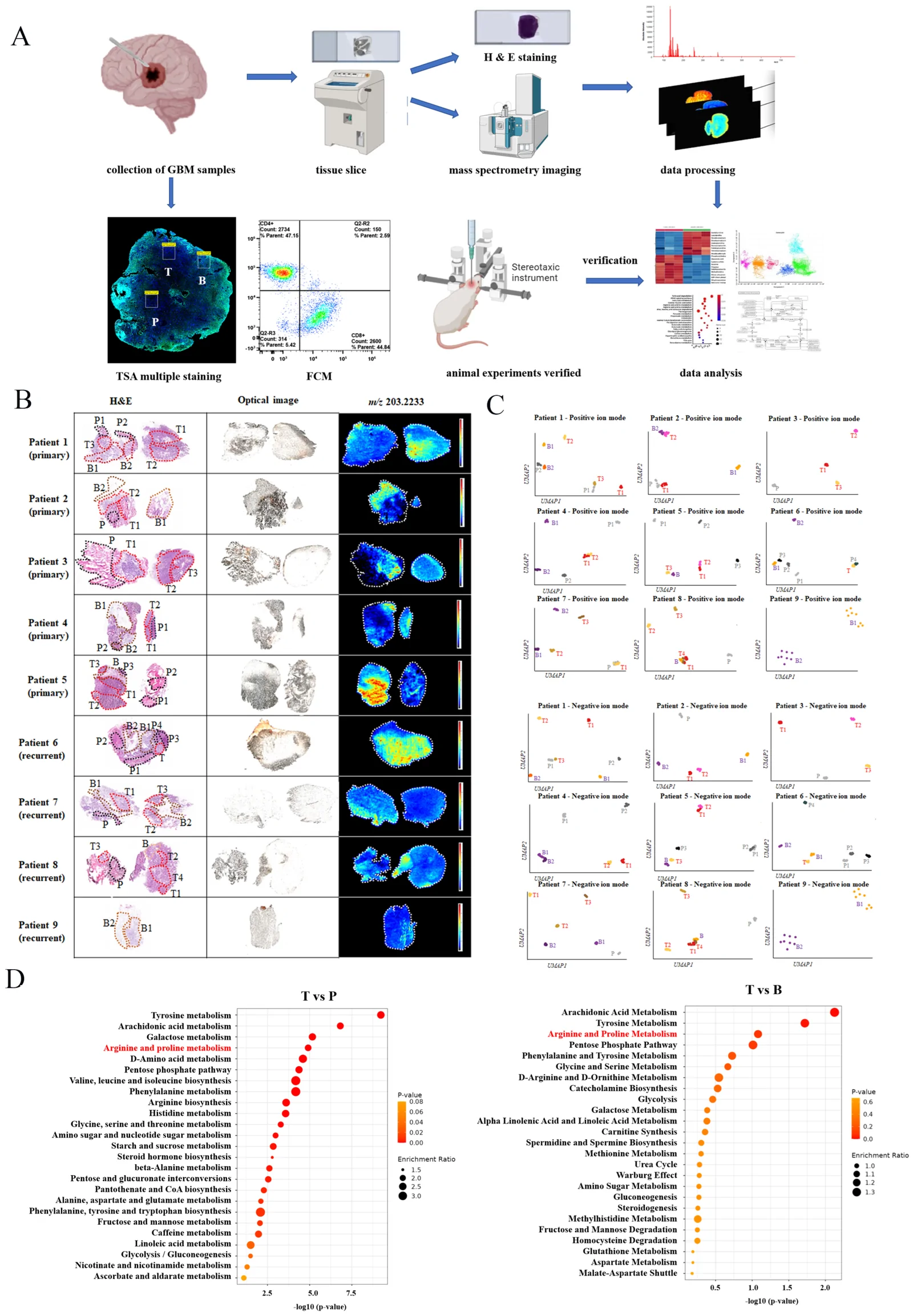
Identification of spatial metabolic characteristics in different pathological regions of GBM. (**A**) Schematic overview of the study design. (**B**) Representative H & E staining, light microscopy, and MSI of SPM (*m*/*z* 203.2233) from the same GBM tissue section (biological patients *n* = 9, including 5 primary patients with GBM and 4 recurrent patients with GBM). SPM intensity shows regional differences among P, T, and B, illustrating the ability of MSI to resolve region-specific metabolic heterogeneity. SPM is shown as an example metabolite and was not used for normalization. All MSI data were normalized using the total ion current (TIC). The vertical color bar represents the signal intensity of the target metabolite in MSI images. Red corresponds to the highest signal intensity, and the color gradually transitions through orange, yellow, green, cyan, blue to dark blue, which indicates the lowest signal intensity. (**C**) UMAP clustering of metabolic profiles in positive ion mode (above) and negative ion mode (below). A total of 5 primary patients with GBM and 4 recurrent patients with GBM were analyzed together to reveal conserved regional metabolic separation across GBM tissues. Regional segmentation of GBM tissues was determined by pathological diagnosis of serial H&E-stained sections (gold standard). UMAP analysis is only used for auxiliary exploratory verification of regional segmentation rationality and does not provide statistical evidence for significant metabolic differences among regions. UMAP clustering may be affected by patient heterogeneity and unbalanced pixel sampling, and no statistical inference was made based on this visualization. (**D**) Pathway enrichment of differential metabolites in different regions of patients with GBM (left: T vs. P; right: T vs. B). Color intensity corresponds to −log_10_(FDR-adjusted *p*-value). Dot size indicates enrichment ratio. Full enrichment statistics and input feature information are provided in Supplementary Tables S3 and S4.

The research approach of the entire study is illustrated in Figure 1A. Tissue sections were classified into P, T, and B regions via H & E staining and matched with MSI (Figure 1B). UMAP clustering showed visual separation of metabolic profiles across different pathological regions in both positive and negative ion modes, serving as an exploratory observation suggestive of spatial metabolic heterogeneity (Figure 1C). Pathway enrichment revealed significant alterations in arginine and proline metabolism across regions (Figure 1D), indicating that changes in arginine and proline metabolism are relatively common in different pathological regions of GBM and may play important roles in the pathological progression of GBM, which we further investigated in this study.

### 3.2. Spatial Reconfiguration of Polyamine Metabolism in GBM

**Figure 2:**
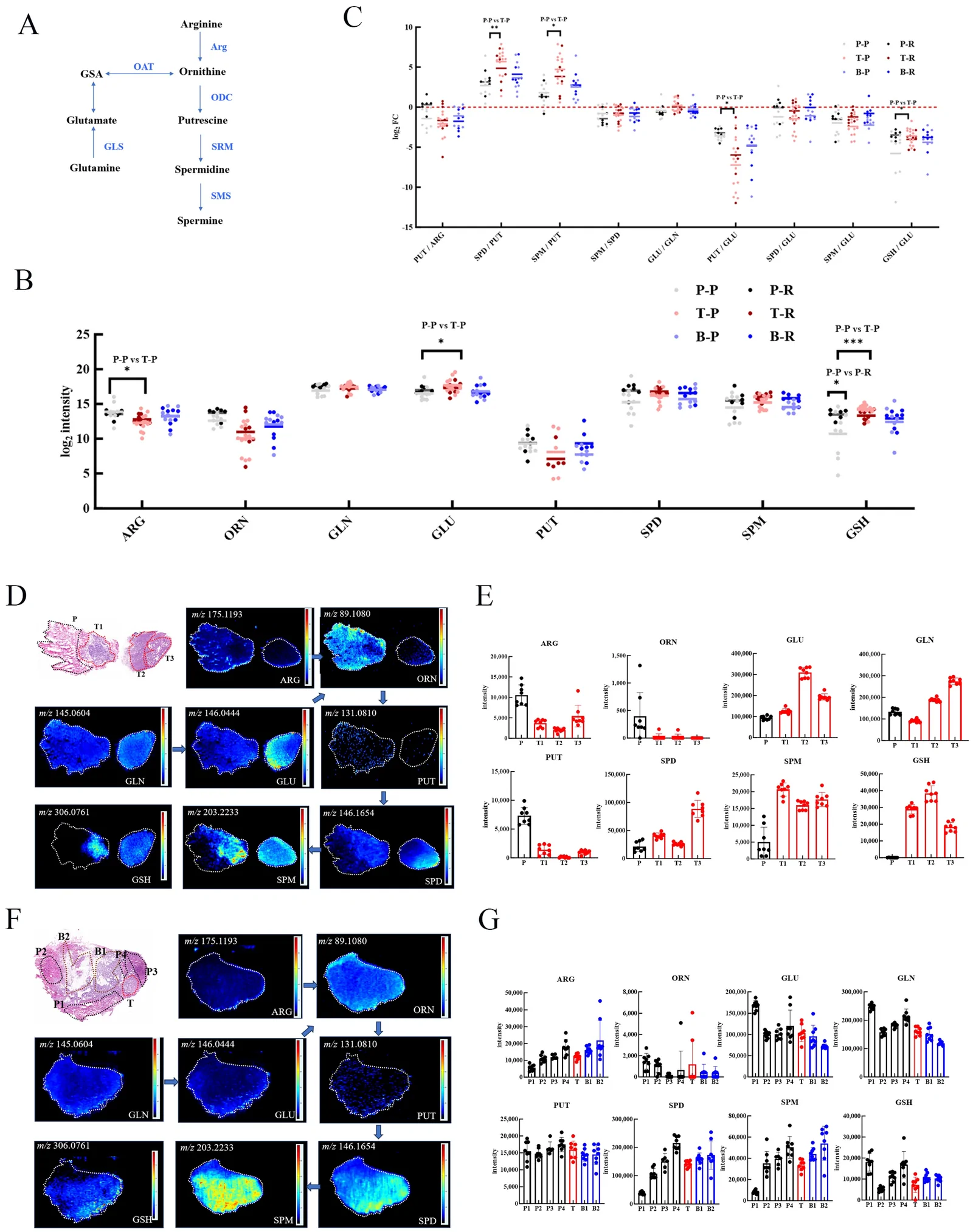
Spatial reconfiguration of polyamine metabolism in GBM. (**A**) Schematic diagram of polyamine-metabolism-related metabolites and metabolic enzymes. (**B**) Distribution of polyamine metabolite intensities (normalized by TIC) in the P, T, and B regions of primary and recurrent GBM (biological patients *n* = 9, including 5 primary patients with GBM and 4 recurrent patients with GBM). The intensity was expressed in logarithmic form. The horizontal line represents the average value. (**C**) Ratio analysis of polyamine metabolites in the P, T and B regions of GBM (biological patients *n* = 9, including 5 primary patients with GBM and 4 recurrent patients with GBM). The ratio is expressed in logarithmic form. Taking the vertical coordinate of 0 as the baseline for the ratio, at this point the ratio is 1, indicating that the intensities of the two metabolites are equal. The horizontal line represents the average value. The red dashed horizontal line indicates the zero baseline for comparison. (**D**,**E**) Spatial distribution and quantitative bar charts of polyamine metabolic metabolites in different pathological regions of a primary patient with GBM (patient ID: 3). (**F**,**G**) Spatial distribution and quantitative bar charts of polyamine metabolites in different pathological regions of a recurrent patient with GBM (patient ID: 6). Individual data points are represented by colored dots. Black dots represent for P regions, red dots represent for T regions, and blue dots represent for B regions. Horizontal solid lines denote the mean value for each group, with error bars representing the standard error of the mean. Data are presented as mean ± SD. Biological patients *n* = 9 (core cohort), with 2 tissue sections per patient used for detection in both positive and negative ion modes. Each section was divided into several regions (P, T, B) based on H&E staining and 8 pixels per region. Pixels and regions of interest are technical observations, not independent biological replicates. No statistical inference is performed on these single-patient examples; all statistical assessments are derived from the full nine-patient cohort shown in panels (**B**,**C**). Significance markers and F-statistics are only reported for panels (**B**,**C**). For regional comparisons (P, T, B) within the same patient, statistical significance was assessed by mixed-effects models with patient as a random effect. The F values and degrees of freedom for each statistically significant comparison are as follows. (**B**) (ARG: F (5, 42) = 2.290, GLU: F (5, 42) = 2.365, F (5, 42) = 5.022). (**C**) (SPD/PUT: F (5, 41) = 4.026, SPM/PUT: F (5, 39) = 4.187, PUT/GLU: F (5, 42) = 3.088, GSH/GLU: F (5, 44) = 2.430). * *p* < 0.05, ** *p* < 0.01, *** *p* < 0.001.

The polyamine metabolites and related metabolic enzymes are shown in Figure 2A. The intensities of the polyamine metabolites in the P, T, and B pathological microregions in primary and recurrent GBM (Figure 2B) and the intermediate metabolite ratio were analyzed across different pathological microregions (Figure 2C). The expression intensities and quantitative analyses of polyamine metabolites in different regions of primary and recurrent GBM were presented in one case each (Figure 2D–G). The results showed that polyamine metabolism in GBM is characterized by a reconfiguration of spatial position.

In primary and recurrent GBM, T regions accumulated high levels of spermidine (SPD) and spermine (SPM), with elevated SPD/PUT and SPM/PUT ratios compared with the B and P regions. Upstream arginine (ARG), ornithine (ORN), and putrescine (PUT) were downregulated in T regions compared with the B and P regions, while glutamate (GLU)/glutamine (GLN) was upregulated in T regions compared with the B and P regions, showing spatial coincidence with elevated SPD and SPM levels. GSH and GSH/GLU levels were elevated in the T regions compared with the B and P regions.

In summary, different regions of GBM exhibit differential regulation of polyamine metabolism. In both primary and recurrent patients, the T regions exhibit higher putatively annotated MSI signals for metabolites within the GLN-GLU bypass, accompanied by elevated SPD and SPM signals, whereas the contents of SPD and SPM in the P and B regions are relatively lower. This distinct spatial distribution of polyamine-associated MSI signals represents a spatial association, and any potential biological relevance requires further functional validation.

### 3.3. Analysis of Lipid-Peroxidation Status and Immune Microenvironment in Different Regions of GBM

Multiplex immunofluorescence co-staining was performed on the primary and recurrent tissues of patients with GBM to detect the expression of the classic biomarker of ferroptosis, 4-hydroxynonenal (4-HNE). Combined with the results of the H & E staining of the tissues, the regional expression of 4-HNE in GBM was analyzed.

**Figure 3:**
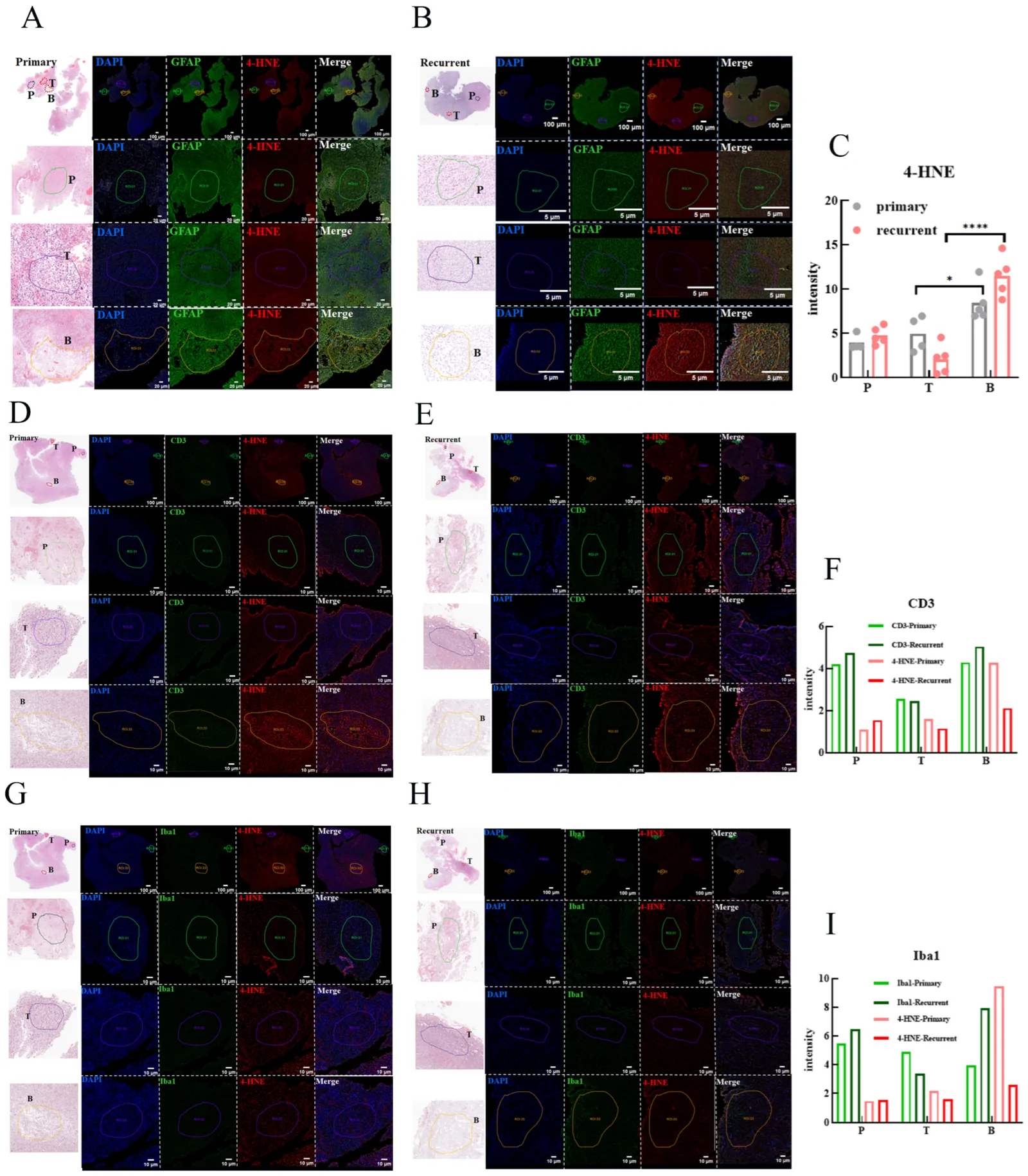
Regional distribution of lipid-peroxidation-associated signals in the primary and recurrent patients with GBM. (**A**–**C**) The regional expression and quantitative analysis of 4-HNE in the primary and recurrent patients with GBM (biological patients *n* = 10, including five primary patients with GBM and five recurrent patients with GBM); paraffin sections were selected for the experiment. The staining conditions of two typical patients are shown in A and B, and the quantitative staining results of the 10 patients are presented in C. The quantification presented in Figure 3C integrates all patient specimens shown in Figure 3A,B, Figures S1 and S2. Representative images in Figures S1 and S2 are additional patient cases included in this quantitative analysis and were moved to the Supplementary Materials due to main-figure layout constraints. Red represents 4-HNE, green represents GFAP, and blue represents DAPI. Valid sample sizes for each pairwise comparison: P versus T (*n* = 9 patients), T versus B (*n* = 9 patients). Statistical significance was assessed by mixed-effects models with patient as a random effect. The F values and degrees of freedom for each statistically significant comparison are as follows: primary (F (2, 10) = 8.246), recurrent (F (2, 12) = 41.09). * *p* < 0.05, **** *p* < 0.0001. (**D**–**F**) The co-dyeing and quantitative analysis of different regions of CD3 and 4-HNE in the primary and recurrent patients with GBM (biological patients *n* = 2; primary patient ID: 2, recurrent patient ID: 8). Red represents 4-HNE, green represents CD3, and blue represents DAPI. No statistical testing was performed due to the single-patient sample and these observations cannot be generalized to the full patient cohort. (**G**–**I**) The co-dyeing and quantitative analysis of different regions of Iba1 and 4-HNE in the primary and recurrent patients with GBM (biological patients *n* = 2; primary patient ID: 2, recurrent patient ID: 8). Red represents 4-HNE, green represents Iba1, and blue represents DAPI. No statistical testing was performed due to the single-patient sample and these observations cannot be generalized to the full patient cohort.

The results revealed that 4-HNE expression was relatively high in the B region of GBM tissues but relatively weak in the P and T regions in both primary and recurrent patients. Based on this regional distribution of 4-HNE, we infer that the B region of GBM showed a higher state of lipid peroxidation, while the P and T regions showed lower 4-HNE expression, consistent with a lower lipid-peroxidation state (Figure 3A–C).

To further investigate the ferroptosis status of immune cells in primary and recurrent patients with GBM, we performed multiplex immunofluorescence co-staining of 4-HNE with immune-cell markers in a limited number of specimens (CD3 as a biomarker of lymphocytes, Iba1 as a biomarker of resident microglia and infiltrating macrophage populations). Both CD3+ and Iba1+ cells exhibited a similar regional pattern. Lipid-peroxidation-associated 4-HNE signals occur in lymphocytes and microglia within GBM and the degree of 4-HNE in the B regions is greater than that in the T region in both primary and recurrent patients (Figure 3D–I).

**Figure 4:**
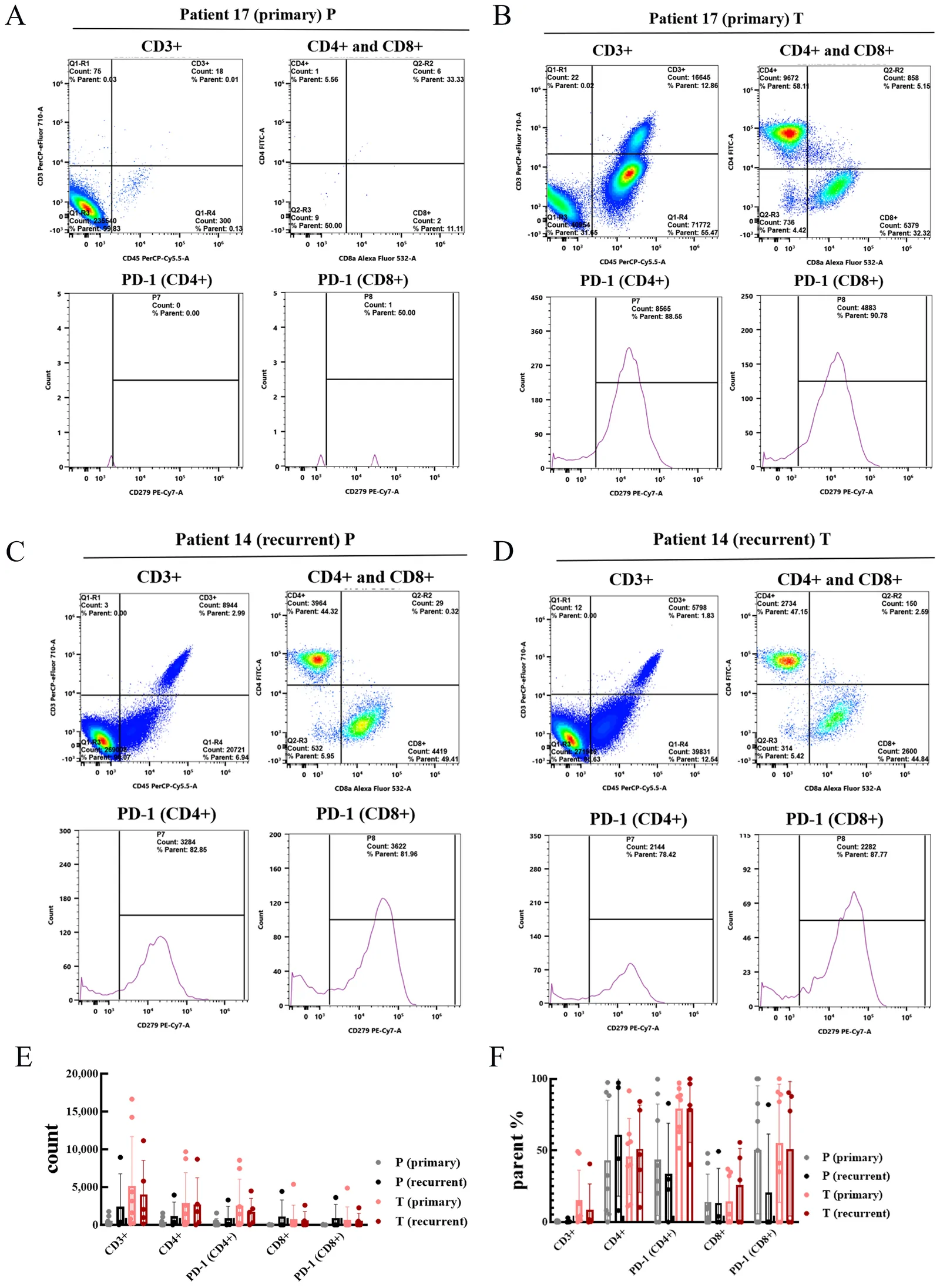
Analysis of the immune microenvironment of GBM. (**A**,**B**) Immunological microenvironmental analysis by FCM of P and T tissues of primary patients with GBM. (**C**,**D**) Immunological microenvironmental analysis by FCM of P and T tissues of recurrent patients with GBM. (**E**,**F**) Analysis of the count and proportion of immune cells of 8 primary patients and 5 recurrent patients. The data used in this analysis show no statistically significant differences among groups (this experiment included fresh tissues from 8 primary patients and 5 recurrent patients; patient 17 and patient 14 were selected for presentation). The color gradient represents the density of events in this flow cytometry contour/density plot: red indicates the highest event density, followed by yellow, green, and blue; blue corresponds to the lowest density of detected cell events.

Therefore, we used flow cytometry to compare the immune-cell landscape and PD-1 marker expression profiles between P tissue and T tissue separated manually before dissociation of patients with GBM. The flow cytometry results of patients with GBM revealed that the number and proportion of CD3+ cells in the P tissue were extremely low and suggested a relatively weak immune microenvironment in the P region of GBM. In GBM tumor tissues, a numerical trend of altered immune-cell counts, including CD8+ cells, was observed, although no statistically significant regional differences were detected, accompanied by detectable PD-1 expression on both CD4+ and CD8+ T cell subsets (Figure 4A–F; Table S2). Notably, PD-1 expression alone cannot serve as definitive evidence of T cell exhaustion, as it is also induced during physiological T cell activation. Combined with substantial inter-patient variability and several immune subsets quantified from very low cell counts in our dataset, these results only reflect altered PD-1-associated immune phenotypic characteristics in tumor tissues. No statistically significant regional differences were observed, and we refrain from overinterpreting these trends as confirmed immune exhaustion.

In summary, lipid-peroxidation state within GBM shows regional specificity. The B region shows a higher state of lipid peroxidation, while the T region shows a lower state of lipid peroxidation. In addition, we observed regional lipid-peroxidation signals adjacent to infiltrating immune cells and variable PD-1-related immune phenotypic alterations within tumor tissues. These descriptive spatial correlations provide preliminary clues for region-specific oxidative stress and immune heterogeneity in GBM, yet the causal relationship linking lipid peroxidation and immune phenotypic changes to the immunosuppressive microenvironment remains to be further functionally validated.

### 3.4. Spatial Associations Between Polyamine-Metabolism-Related Signatures and Lipid-Peroxidation-Associated Signals Across GBM Microregions

**Figure 5:**
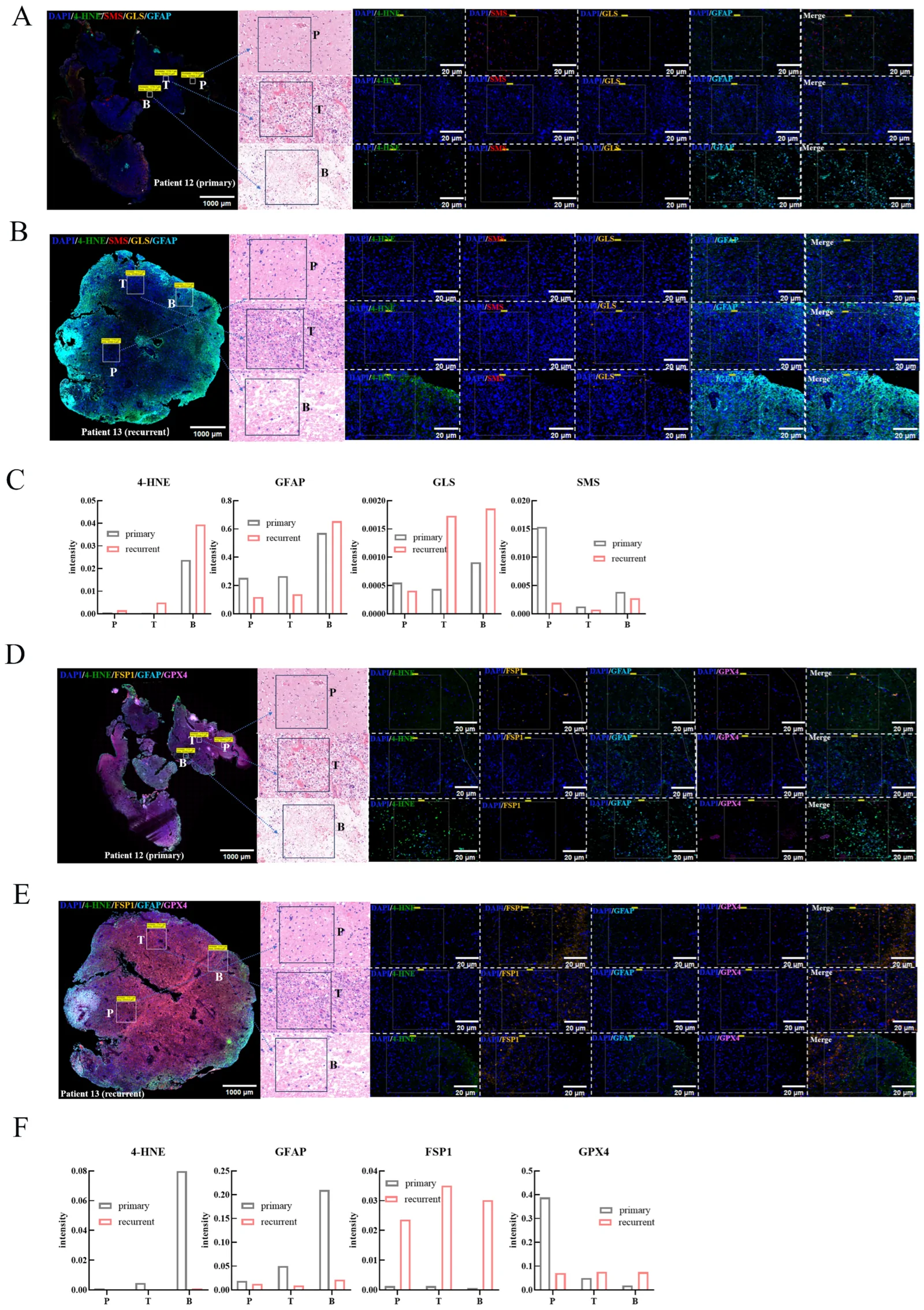
Analysis of the association between polyamine-metabolism-related signatures and lipid-peroxidation-associated signals in different regions of GBM. (**A**–**C**) The regional expression and quantitative analysis of polyamine metabolic enzymes and 4-HNE of primary and recurrent patients with GBM (biological patients *n* = 1; patient 12 and patient 13 were selected for this experiment, representing a paired case of primary and recurrent patients with GBM). No statistical testing was performed due to the single-patient sample. (**D**–**F**) The regional expression and quantitative analysis of ferroptosis-related proteins in primary and recurrent patients with GBM (biological patients *n* = 1; patient 12 and patient 13 were selected for this experiment, representing a paired case of primary and recurrent patients with GBM). No statistical testing was performed due to the single-patient sample and these observations cannot be generalized to the full patient cohort.

Regional heterogeneity in polyamine abundance and lipid-peroxidation-associated signatures exists within GBM tissues, and these two sets of spatial features appear closely linked. To explore spatial patterns of the polyamine–ferroptosis axis, we performed multiplex immunofluorescence co-staining for polyamine-related metabolic enzymes (SMS and GLS) together with the lipid-peroxidation marker 4-HNE on primary and recurrent matched tissue specimens from the same GBM patient (Figure 5A–C). Notably, SMS acts as a core polyamine-synthesizing enzyme, whereas GLS is primarily a glutaminase with only indirect relevance to polyamine metabolism.

Our multiplex immunofluorescence results revealed descriptive regional co-occurrence trends between enzyme expression and lipid-peroxidation-associated signals in GBM. In both primary and recurrent patients, stronger GLS was observed in the B region relative to the T region, where GLS signals spatially overlapped with elevated 4-HNE staining. Similarly, SMS exhibited higher signal intensity in the B region compared with the T region and also spatially co-localized with 4-HNE. These observations are descriptive spatial patterns derived from a limited number of specimens and do not represent formal patient-level correlation or confirmed mechanistic links (Figure 5C).

We conducted immunofluorescence co-staining for multiple ferroptosis-suppressor proteins (FSP1, GPX4) and 4-HNE in the tumor tissues of the same patient with GBM at the primary and recurrent stages to explore the regulatory mechanism through which GBM affects ferroptosis (Figure 5D–F). In both the primary and recurrent GBM patients, the expression of FSP1 and GPX4 in the T region was stronger than that in the B region, and this exhibited an opposite spatial pattern relative to 4-HNE staining intensity. These are descriptive imaging observations from one paired primary-recurrent patient specimen, without formal statistical correlation analysis.

Glutathione (GSH) is a necessary substrate for the ferroptosis-inhibitory effect exerted by GPX4, and xCT can mediate cysteine uptake to synthesize GSH [16,17]. The results of spatial metabolomics revealed that GSH relatively accumulated in the T region of GBM compared with the B region (Figure 2B), which might support GPX4-mediated ferroptosis resistance in the T region.

In summary, descriptive spatial co-occurrence between polyamine-related metabolic features and lipid-peroxidation-associated signatures can be seen across GBM microregions. SMS and GLS show spatial overlap with 4-HNE. The ferroptosis-regulatory proteins GPX4-GSH and FSP1 also display heterogeneous regional distribution. These markers may serve as auxiliary readouts hinting at divergent oxidative-stress features: reduced lipid-peroxidation-associated signals in the T region and elevated signals in the B region. Further functional work is required to validate these descriptive findings.

### 3.5. Reconstruction of Polyamine Metabolism: Partial Recapitulation and Exploration at the Animal Level

**Figure 6:**
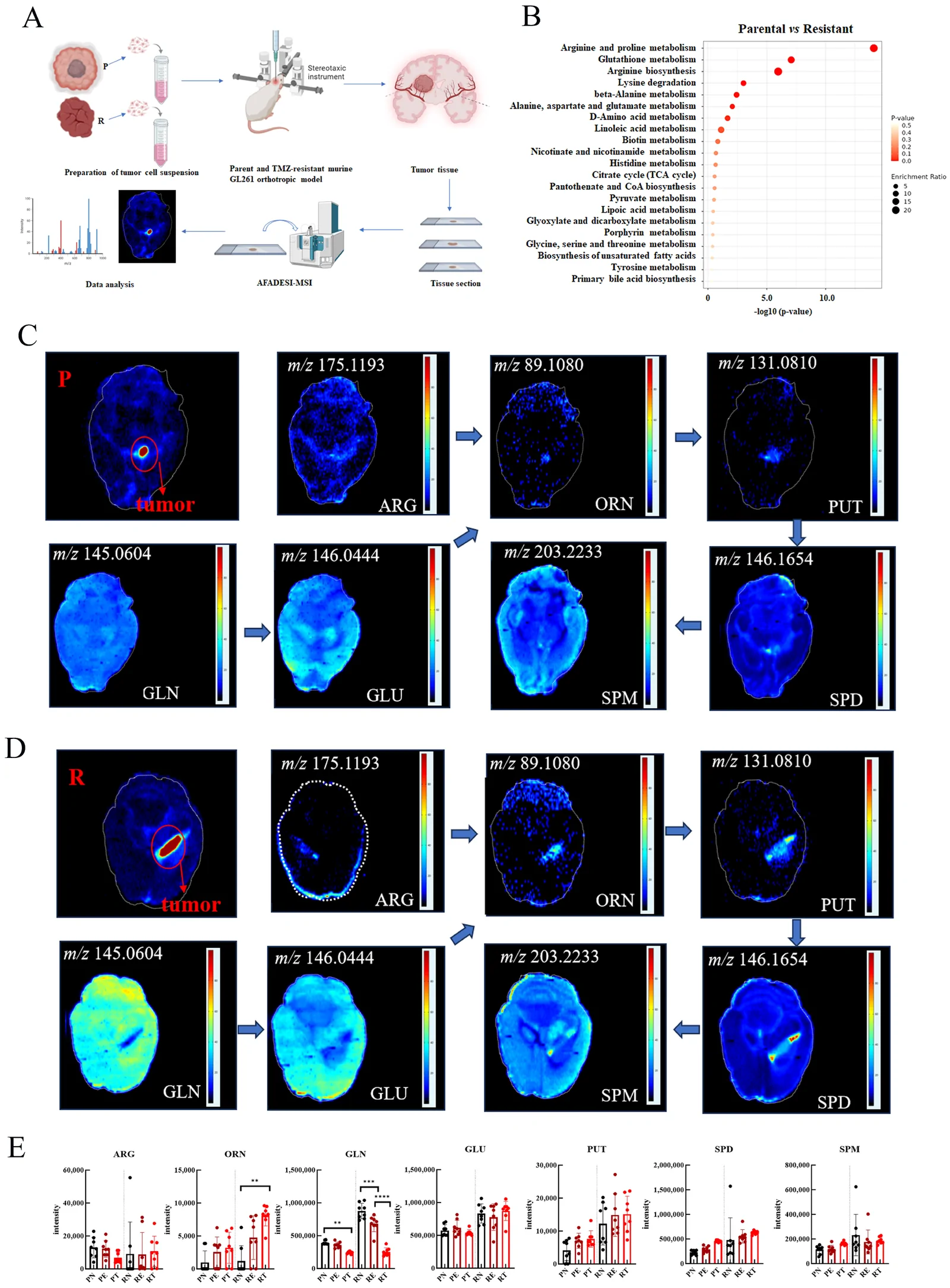
Reconstruction of polyamine metabolism: partial recapitulation and exploration at the animal level. (**A**) Experimental workflow for parental and TMZ-resistant GL261 orthotopic models. (**B**) Enrichment of metabolic pathways showing differences between parental and tumor cells derived from the in vivo TMZ-selected GL261 line groups of animals. Pathway enrichment compares aggregated metabolite profiles from tumors derived from parental GL261 versus tumors derived from the in vivo TMZ-selected GL261 line. Differential features used for enrichment were calculated from mouse-level aggregated values instead of individual pixel-level observations. Full enrichment statistics are provided in Supplementary Table S5. (**C**–**E**) Spatial distribution and quantitative diagrams of polyamine-metabolism-related metabolites in different pathological regions of mice in parental and tumor cells derived from the in vivo TMZ-selected GL261 line groups. The biological repetition was *n* = 3. A single representative image is shown for each group, selected based on pre-defined objective histopathological criteria (sufficient tumor size, intact whole-brain tissue section, unambiguous stereotactic tumor engraftment). Eight sampling points were chosen as technical measurement units for each region. Multiple spatial regions sampled within an individual mouse brain represent intra-sample technical observations and are not treated as independent biological replicates for statistical comparison. Statistical significance was assessed by mixed-effects models with mouse as a random effect. The F values and degrees of freedom for each statistically significant comparison are as follows: E (ORN:F (5, 42) = 10.29, GLN:F (5, 42) = 88.00). ** *p* < 0.01, *** *p* < 0.001, **** *p* < 0.0001.

The constructed GL261 parental and tumor cells derived from the in vivo TMZ-selected GL261 line were orthotropically transplanted into the brains of mice. Spatial metabolomics was performed on the orthotropic implanted tumors to partially recapitulate the metabolic changes in the GBM of the parental and tumor cells derived from the in vivo TMZ-selected GL261 line mice at the animal level (Figure 6A). The results of pathway enrichment showed that arginine and proline metabolism remained the most significantly different both in the parental and tumor cells derived from the in vivo TMZ-selected GL261 line groups, which were consistent with those at the patient level of GBM (Figure 6B).

The parental and tumor cells derived from the in vivo TMZ-selected GL261 line tissue sections were divided into three regions: normal tissue (PN, RN), para-carcinoma tissue (PE, RE), and tumor tissue (PT, RT). The metabolites related to polyamine metabolism were analyzed and quantified through image analysis (Figure 6C–E). ARG is mainly downregulated in the T region of the parental group, while ORN shows a gradient increase in the N, E, and T regions between the two groups. We infer that the upregulation of ORG expression was more likely to result from the degradation of ARG. GLN showed a gradient decrease in the N, E, and T regions across both groups, while the within-group changes in GLU were not significant. Among the polyamines, PUT and SPD tended to increase from the N to E to T tissues both in the parental and tumor cells derived from the in vivo TMZ-selected GL261 line groups (Figure 6C–E). The distribution of SPD showed a similar trend in both mice and patients, whereas the distribution of PUT showed an opposite trend between mice and patients (Figure 2D–G). Of note, PUT exhibited opposite regional trends between mouse GL261 tumors and human GBM specimens, whereas SPD showed comparable regional enrichment. Therefore, the GL261 orthotopic model only partially recapitulates human GBM spatial polyamine signatures and cannot fully reproduce all metabolic features observed in patient tissues [18].

On the basis of the above results, similarly to those of patients with GBM, the polyamine metabolites also exhibited regional reorganization at the mouse level. The levels of ORN, PUT and SPD increase in a gradient pattern in the N, E and T regions, and they are mainly enriched in the T region both in the parental and tumor cells derived from the in vivo TMZ-selected GL261 line groups.

## 4. Discussion

Through the integration of spatial metabolomics analyses and biological validation, this study provides a spatially resolved characterization of the polyamine–ferroptosis axis in GBM, revealing the spatial characteristics of the polyamine-metabolism and ferroptosis states within GBM, as well as the spatial correlations between them.

Spatial heterogeneity is a hallmark of GBM progression, and region-specific metabolic rewiring enables tumor adaptation to the microenvironment. Prior work highlighted arginine–proline metabolism in GBM proliferation and invasion, yet bulk analyses could not resolve spatial patterns [19,20,21]. In this study, through the precise matching of mass spectrometry imaging and pathological morphology, the metabolic microdomains of the P, T, and B regions in GBM were clearly defined in the spatial dimension. Nevertheless, the current study treats all annotated necrotic areas predominantly as geographic necrosis. Small pseudopalisading necrotic foci were not analyzed as independent subgroups. Future spatial metabolomics studies with dedicated necrosis-subtype segmentation may uncover subtype-specific metabolic signatures. UMAP clustering confirmed distinct metabolic profiles for each region, validating spatial metabolomics as a robust tool to dissect intratumoral metabolic heterogeneity and revealing consistent dysregulation of the arginine–proline pathway across regions.

In previous studies, the orthotopic brain transplantation model has been widely used in the research of GBM metabolic reprogramming and treatment resistance [22,23], but verification of the spatial regulation of the polyamine-metabolism–ferroptosis axis in animal models has not been reported. In this study, using orthotopic brain tumor models derived from parental and in vivo TMZ-selected GL261 cells, we observed spatial alterations in polyamine-associated MSI signals in mice. Some of these spatial patterns showed partial similarity to those seen in human GBM specimens. Elevated SPD-associated MSI signals were detected within mouse tumor regions, and altered spatial MSI signals for metabolites of the GLN-GLU metabolic axis were observed in tumors from the in vivo TMZ-selected GL261 line. In summary, the animal experiments provided an experimental basis and model for translational application of the polyamine-metabolism–ferroptosis axis as a therapeutic target for GBM. However, the TMZ-resistant GL261 model used in this study was generated by repeated in vivo TMZ exposure over several passages, which induces adaptive metabolic changes but cannot replicate the years-long clonal evolution and multiple rounds of therapy that characterize human recurrence. It should be noted that not all polyamine spatial patterns are consistent between GL261 mouse tumors and human GBM. Putrescine displays opposing regional distribution trends across species, while spermidine shows similar tumor-region enrichment. This divergence may stem from composite factors: inter-species differences in polyamine-metabolizing enzyme regulation; large differences in necrotic-zone scale and immune–stromal composition; and distinct selective pressures between rapidly growing untreated mouse tumors and long-evolved, therapy-exposed human clinical GBM. Our current data cannot pinpoint the exact mechanistic driver for this discrepancy, which represents a key translational limitation of this orthotopic model. We acknowledge that repeating MSI profiling on patient-derived orthotopic xenograft models with humanized immune reconstitution would deliver more physiologically faithful translational evidence of the polyamine–ferroptosis spatial axis in recurrent GBM. Such extended animal validation will be prioritized in our subsequent mechanistic studies to establish causal functional links between polyamine rewiring and ferroptosis resistance, building upon the correlative spatial framework established in the present clinical and GL261-based dataset.

It is also worth noting that the immunocompetent C57BL/6J GL261 orthotopic glioma model cannot fully recapitulate the immune microenvironment of human GBM. Human glioblastoma is characterized by abundant monocyte-derived suppressive tumor-associated macrophages, sparse tumor-infiltrating T cells, and complex myeloid-mediated immune-suppressive networks. In contrast, GL261 tumors in C57BL/6J mice exhibit distinct myeloid polarization and lymphocyte-recruitment features. These inter-species immune discrepancies may reshape local polyamine-metabolism- and lipid-peroxidation-associated signals within tumor microregions [24,25]. Hence, metabolic spatial patterns observed in mouse tumors should be interpreted with caution and cannot be directly extrapolated to clinical human glioblastoma.

Polyamines exert dual regulation on ferroptosis: SPD and SPM suppress ferroptosis via ROS scavenging, while acetylated polyamines promote ROS-driven lipid peroxidation and ferroptosis [26,27]. Moreover, the imbalance in ferroptosis regulation in tumors has been confirmed to be closely related to the malignant progression and treatment resistance of GBM [8,28]. However, these studies have not clarified the differences in the distribution of polyamine metabolites in different pathological regions of GBM or their regional regulatory effects on ferroptosis. We observed distinct spatial associations of polyamine-related MSI-detected signals across different glioblastoma microregions both in primary and recurrent GBM patients: T regions exhibited higher SPD and SPM-associated MSI signals together with lower lipid-peroxidation-associated 4-HNE signals, whereas B regions showed relatively lower polyamine-associated signals and elevated 4-HNE signals. Because AFADESI-MSI provides static spatial abundance rather than dynamic flux information, our observations describe spatial associations rather than causal or flux relationships. Metabolite ratios such as SPD/PUT and SPM/PUT were used as descriptive indicators of relative regional abundance; they do not directly demonstrate biochemical interconversion, as ionization efficiencies may differ across metabolites. Establishing true metabolic flux will require isotope-tracing experiments or other direct measurements of pathway activity, which represent an important direction for future investigation. It also should be noted that elevated MSI signals of SPD and SPM do not exclusively indicate enhanced de novo polyamine synthesis. Polyamines are strongly cationic and dynamically bind to DNA, RNA, chromatin, cellular membranes and proteins. Regional signal changes may arise from multiple alternative biological processes: shifts in the equilibrium between macromolecule-bound and mobile polyamine pools, altered cellular uptake or export capacity, changes in polyamine catabolism mediated by SAT1, SMOX and PAOX, heterogeneity in local cellular density, and polyamine release from damaged or necrotic tumor cells. Since we have not performed enzyme-activity measurements for ODC1 or SRM, isotope-tracing experiments, uptake-export functional assays, or separation of free versus bound polyamine fractions, the precise origin of spatially increased spermine and spermidine cannot be resolved in this exploratory study. Future functional investigations will be necessary to distinguish these competing possibilities.

It is important to distinguish polyamine abundance from polyamine catabolic activity, as these two states can exert opposite effects on ferroptosis. Intact spermine and spermidine possess radical-scavenging antioxidant properties and may mitigate lipid peroxidation. In contrast, polyamine catabolism mediated by SAT1 and polyamine oxidases (SMOX, PAOX) generates hydrogen peroxide and reactive aldehydes, which can amplify oxidative stress and promote ferroptosis. Therefore, elevated polyamine abundance does not necessarily indicate elevated polyamine catabolic activity, and the net effect on ferroptosis depends on the balance between intact polyamine pools and their catabolic turnover. In our study, the spatial co-occurrence of high spermine/spermidine with low 4-HNE in tumor regions is consistent with a potential antioxidant effect of intact polyamines. However, this observation does not establish that polyamines confer ferroptotic resistance, as regional differences may alternatively reflect shifts in polyamine binding to nucleic acids or proteins, altered turnover, or other processes. The proposed connection between polyamine metabolism and ferroptosis should be treated as a hypothesis requiring functional confirmation. Our static MSI dataset does not include direct measurements of SAT1, SMOX or PAOX activity, free-versus-bound polyamine fractions, or functional ferroptosis assays; these experiments will be essential in future work to resolve the direction and magnitude of the polyamine–ferroptosis relationship.

Notably, the T region harbored higher MSI signals of GLN and GLU, whereas the B region showed relatively stronger GLS immunofluorescence intensity, resulting in an apparent spatial mismatch between metabolite abundance and enzyme protein expression. Several microenvironmental factors may potentially account for this inconsistent pattern. First, GLS immunofluorescence only reflects relative protein abundance and may not necessarily correspond to actual catalytic metabolic flux. It is speculated that the elevated GLS protein signal in the B region may arise from stress-induced responses in inflammatory or reactive glial cells within necrotic microenvironments, rather than reflecting tumor-intrinsic glutaminolysis activity. In addition, extensive tissue disintegration and metabolite diffusion in the B region may potentially lead to the loss and reduced retention of GLN/GLU metabolites, even when local GLS protein levels are relatively high. In contrast, viable tumor cells in the T region may actively uptake and preserve glutamine-related metabolites to support local biosynthetic programs, which could contribute to the relatively higher GLN and GLU MSI signals despite moderate GLS staining. Collectively, these speculative microenvironmental differences might partially explain the observed decoupling between enzyme expression and metabolite distribution, although further functional validation is required to confirm these mechanisms.

Beyond the GSH-GPX4 and FSP1 antioxidant systems, multiple additional molecules participate in ferroptosis regulation within glioblastoma. The cystine–glutamate antiporter xCT (SLC7A11) controls cysteine import for GSH biosynthesis and ACSL4 contributes to the generation of polyunsaturated fatty-acid-containing phospholipids that are susceptible to lipid peroxidation. DHODH represents another important inner-mitochondrial ferroptosis suppressor, while GCH1 can counteract ferroptosis via tetrahydrobiopterin-mediated lipid peroxide scavenging. Collectively, these regulators shape the sensitivity of tumor cells toward lipid-peroxidation-driven cell death [29,30]. It should be noted that our present dataset lacks direct spatial protein or transcript evidence for xCT, ACSL4, DHODH and GCH1 in GBM microregions; further multiplex spatial profiling will be required to dissect their regional contribution.

Furthermore, polyamine metabolism exerts context-dependent dual effects on ferroptosis. Unmodified spermidine and spermine can function as radical-scavenging antioxidants to mitigate lipid peroxidation. In contrast, acetylated polyamines, produced via polyamine acetylation, are substrates for catabolic enzymes such as SAT1 and PAOX. Their breakdown generates hydrogen peroxide and reactive aldehydes, which can amplify oxidative stress and promote lipid peroxidation [12]. Such opposing functions mean that simply detecting high regional SPD/SPM abundance cannot distinguish between antioxidant-protective effects and downstream pro-ferroptotic catabolic turnover. This complexity reinforces that our spatial metabolomics observations remain correlative, and functional assays are necessary to resolve how polyamine redistribution shapes ferroptosis-related phenotypes in distinct GBM regions.

Correlative spatial observations show lipid-peroxidation-associated signals localizing near tumor-infiltrating immune-cell populations. Conventional mechanistic frameworks for GBM immune suppression predominantly center on T cell exhaustion mediated by immune checkpoint molecules such as PD-1, together with M2-polarized microglia and myeloid-derived suppressor cell enrichment, which collectively obstruct anti-tumor cytotoxic function [31,32]. However, the present study provides in situ spatial evidence that there are lipid-peroxidation-associated signals present in CD3^+^ and Iba1^+^ cells across the T and B regions. This observation complements and expands existing models of GBM immune evasion, where effector immune populations displayed phenotypic signs of dysfunction, and lipid-peroxidation-associated 4-HNE signals were detected in their vicinity. These correlative observations cannot formally prove immune-cell ferroptosis and require further functional validation.

It is worth noting that even if polyamine–ferroptosis-related signals are markedly altered within the necrotic core of GBM, therapeutic intervention targeting this region faces well-recognized practical delivery obstacles. The necrotic compartment exhibits collapsed tumor vasculature, high interstitial fluid pressure, abundant cellular debris, and severe hypoxia, which collectively restrict the penetration and effective accumulation of systemic small-molecule drugs, biological agents, and nanocarriers. Although our study identifies correlative spatial metabolic patterns in necrotic regions, we do not propose immediate therapeutic translation from these observational findings. Any future strategy aiming to modulate polyamine-dependent ferroptosis inside necrotic foci will need to carefully consider these delivery-related bottlenecks.

This study has several limitations.

Firstly, we acknowledge that most findings from this study are correlative; accordingly, the mechanistic models proposed herein should be regarded as hypotheses requiring functional validation via gene knockout, pharmacological inhibition, or metabolite supplementation experiments in suitable model systems to prove causality.

Secondly, the current study is limited by the moderate size of our patient cohort (total of 17 GBM patients, among which only 6 are recurrent cases). Our study is based on a diagnostically heterogeneous high-grade glioma cohort, which includes WHO grade 3 and H3K27M-mutant cases. Relevant analyses should be interpreted as exploratory. Definitive conclusions restricted strictly to classical IDH wild-type GBM will require future cohorts with complete WHO-integrated molecular classification. In addition, several co-staining analyses (Figure 3D–I and Figure 5A–F) were performed on only one patient specimen. These data can only visualize spatial colocalization patterns as descriptive examples. Larger patient cohorts are required to confirm whether such molecular spatial features are broadly reproducible in GBM. Furthermore, although IDH mutation status was documented and used for case filtering, complete pre-surgical treatment history and MGMT promoter-methylation data are unavailable for this archival patient cohort. These clinical and molecular factors are known to modulate tumor-metabolism- and ferroptosis-related signatures, and their confounding effects cannot be excluded in our present analysis. Future prospective spatial metabolomics studies with comprehensive clinical and molecular metadata will be needed to disentangle these influences. Group-level differences between primary and recurrent tumors may be confounded by inter-patient variability, molecular subtypes, tumor location, sample-handling artifacts, and prior therapies, including temozolomide, radiotherapy, corticosteroids and anti-angiogenic agents. Therefore, we cannot definitively assign inter-group differences to tumor recurrence. Paired patient-matched longitudinal samples will be required in future work to dissect molecular alterations driven by tumor relapse.

Thirdly, it should be noted that all core metabolites supporting our spatial observations (SPD, SPM, PUT, GLU, GLN, GSH) are putatively annotated metabolites from AFADESI-MSI data, based on 5 ppm accurate-mass matching and isotope-pattern assessment. No on-tissue MS/MS or authentic-standard validation was completed on GBM specimens in this work. External LC-MS/MS validation from other human tissues cannot fully rule out interference from isobaric or structurally related compounds. Confidence intervals for metabolite assignments are not provided. Our downstream interpretations are built upon these putative annotations.

Fourthly, although reduced TMZ responsiveness was observed in subcutaneous tumors generated from these cells, key characterizations, including in vitro IC_50_, drug-resistance index, stability of resistant phenotype under drug-free conditions, molecular characterization of resistance-related pathways, and experimental validation of sustained TMZ resistance after intracranial orthotopic implantation, are absent in this study. Comprehensive multi-level model characterization will be required in future work to establish the link between temozolomide resistance and spatial metabolic rewiring. Furthermore, the C57BL/6J-GL261 orthotopic mouse model possesses substantial immune-microenvironment differences compared with human GBM, which restricts direct translational generalization of mouse spatial metabolomics findings to clinical patients.

Finally, our study relies heavily on 4-HNE immunofluorescence to evaluate oxidative damage. Since 4-HNE is a non-specific lipid-peroxidation product, it cannot unambiguously confirm ferroptosis. Definitive evidence for regional ferroptosis would require multiple complementary measurements, such as lipid ROS, labile iron evaluation, assessment of ACSL4-SLC7A11-GPX4 axis activity, mitochondrial ultrastructure analysis, or pharmacological rescue using ferroptosis inhibitors. Such orthogonal validations were not feasible using our archived patient tissues and represent key directions for future work.

Consequently, these data should be considered illustrative and hypothesis-generating, and future studies with larger cohorts are required to confirm the spatial patterns statistically.

## 5. Conclusions

This study describes spatially distinct patterns of polyamine-metabolism- and lipid-peroxidation-associated markers across different pathological regions of GBM. Our spatial metabolomics data reveal correlative spatial associations within the polyamine–ferroptosis-related axis linked to regional heterogeneity: the tumor region shows accumulation of SPD and SPM, altered glutamine–glutamate abundance, and upregulation of the GSH-GPX4 and FSP1 antioxidant markers, accompanied by a lower 4-HNE signal, whereas the necrotic region exhibits lower SPD and SPM together with elevated 4-HNE levels. We also detected lipid-peroxidation-associated signals within immune-cell compartments of GBM alongside immune-cell-dysfunction-related phenotypes, which may correlate with the formation of an immunosuppressive microenvironment. We also identified GLS and SMS as candidate targets for future hypothesis-driven studies. Functional validation through genetic or pharmacological interventions is required to establish causality.

## Data Availability

All data are available from the corresponding author upon reasonable request.

## Supplementary Materials

The following supporting information can be downloaded at: https://www.mdpi.com/article/10.3390/biomedicines14092067/s1. Table S1: Patient information; Table S2: Immune cell counts and proportions in patients with primary and recurrent GBM; Table S3: On pathway enrichment (Figure 1D T vs. P); Table S4: on pathway enrichment (Figure 1D T vs. B); Table S5: on pathway enrichment (Figure 6B); Figure S1: The regional expression of the 4-HNE in the primary GBM patients (patient ID: 2, 3, 4, 5, these specimens are included in the quantitative summary shown in Figure 3C). Patient 4 does not have the typical P region and patient 5 does not have the typical T region. For statistical comparison, only valid pathologically confirmed regions from each individual were included; missing or atypical regions were not imputed, interpolated, or artificially supplemented, and were excluded from corresponding regional pairwise analyses. Red represents 4-HNE, green represents GFAP, and blue represents DAPI; Figure S2: The regional expression of the 4-HNE in the recurrent GBM patients (patient ID: 6, 7, 8, 13, these specimens are included in the quantitative summary shown in Figure 3C). Red represents 4-HNE, green represents GFAP, and blue represents DAPI.

## References

[1] Królikowska K., Błaszczak K., Ławicki S., Zajkowska M., Gudowska-Sawczuk M. (2025). Glioblastoma-A Contemporary Overview of Epidemiology, Classification, Pathogenesis, Diagnosis, and Treatment: A Review Article. Int. J. Mol. Sci..

[2] Sipos D., Raposa B.L., Freihat O., Simon M., Mekis N., Cornacchione P., Kovács Á. (2025). Glioblastoma: Clinical Presentation, Multidisciplinary Management, and Long-Term Outcomes. Cancers.

[3] Ostrom Q.T., Patil N., Cioffi G., Waite K., Kruchko C., Barnholtz-Sloan J.S. (2025). CBTRUS Statistical Report: Primary Brain and Other Central Nervous System Tumors Diagnosed in the United States in 2018–2022. Neuro-Oncol..

[4] Stupp R., Taillibert S., Kanner A., Read W., Steinberg D., Lhermitte B., Toms S., Idbaih A., Ahluwalia M.S., Fink K. (2017). Effect of tumor-treating fields plus maintenance temozolomide vs maintenance temozolomide alone on survival in patients with glioblastoma: A randomized clinical trial. JAMA.

[5] Noorbakhsh A., Tang J.A., Marcus L.P., McCutcheon B., Gonda D.D., Schallhorn C.S., Talamini M.A., Chang D.C., Carter B.S., Chen C.C. (2014). Gross-total resection outcomes in an elderly population with glioblastoma: A SEER-based analysis. J. Neurosurg..

[6] Patel A.P., Tirosh I., Trombetta J.J., Shalek A.K., Gillespie S.M., Wakimoto H., Cahill D.P., Nahed B.V., Curry W.T., Martuza R.L. (2014). Single-cell RNA-seq highlights intratumoral heterogeneity in primary glioblastoma. Science.

[7] Neftel C., Laffy J., Filbin M.G., Hara T., Shore M.E., Rahme G.J., Richman A.R., Silverbush D., Shaw M.L., Hebert C.M. (2019). An integrative model of cellular states, plasticity, and genetics for glioblastoma. Cell.

[8] Dixon S.J., Lemberg K.M., Lamprecht M.R., Skouta R., Zaitsev E.M., Gleason C.E., Patel D.N., Bauer A.J., Cantley A.M., Yang W.S. (2012). Ferroptosis: An iron-dependent form of nonapoptotic cell death. Cell.

[9] Yang W.S., SriRamaratnam R., Welsch M.E., Shimada K., Skouta R., Viswanathan V.S., Cheah J.H., Clemons P.A., Shamji A.F., Clish C.B. (2014). Regulation of ferroptotic cancer cell death by GPX4. Cell.

[10] Chen X., Kang R., Kroemer G., Tang D. (2021). Broadening horizons: The role of ferroptosis in cancer. Nat. Rev. Clin. Oncol..

[11] Stockwell B.R. (2022). Ferroptosis turns 10: Emerging mechanisms, physiological functions, and therapeutic applications. Cell.

[12] Ou Y., Wang S.J., Li D., Chu B., Gu W. (2016). Activation of SAT1 engages polyamine metabolism with p53-mediated ferroptotic responses. Proc. Natl. Acad. Sci. USA.

[13] Li J., Cao F., Yin H.L., Huang Z.J., Lin Z.T., Mao N., Sun B. (2020). Ferroptosis: Past, present and future. Cell Death Dis..

[14] Wang W., Green M., Choi J.E., Gijón M., Kennedy P.D., Johnson J.K., Liao P., Lang X., Kryczek I., Sell A. (2019). CD8+ T cells regulate tumor ferroptosis during cancer immunotherapy. Nature.

[15] Alexandrov T. (2020). Spatial metabolomics and imaging mass spectrometry in the age of artificial intelligence. Annu. Rev. Biomed. Data Sci..

[16] Koppula P., Zhuang L., Gan B. (2021). Cystine transporter SLC7A11/xCT in cancer: Ferroptosis, nutrient dependency, and cancer therapy. Protein Cell.

[17] Robert S.M., Buckingham S.C., Campbell S.L., Robel S., Holt K.T., Ogunrinu-Babarinde T., Warren P.P., White D.M., Reid M.A., Eschbacher J.M. (2015). SLC7A11 expression is associated with seizures and predicts poor survival in patients with malignant glioma. Sci. Transl. Med..

[18] Rizzo G., Bertotti A., Leto S.M., Vetrano S. (2021). Patient-derived tumor models: A more suitable tool for pre-clinical studies in colorectal cancer. J. Exp. Clin. Cancer Res..

[19] Yuxiao C., Jiachen W., Yanjie L., Shenglan L., Yuji W., Wenbin L. (2024). Therapeutic potential of arginine deprivation therapy for gliomas: A systematic review of the existing literature. Front. Pharmacol..

[20] Kuo M.T., Chen H.H.W., Feun L.G., Savaraj N. (2021). Targeting the Proline-Glutamine-Asparagine-Arginine Metabolic Axis in Amino Acid Starvation Cancer Therapy. Pharmaceuticals.

[21] Sawicka M.M., Sawicki K., Jadeszko M., Bielawska K., Supruniuk E., Reszeć J., Prokop-Bielenia I., Polityńska B., Jadeszko M., Rybaczek M. (2024). Proline Metabolism in WHO G4 Gliomas Is Altered as Compared to Unaffected Brain Tissue. Cancers.

[22] Abdelwahab M.G., Sankar T., Preul M.C., Scheck A.C. (2011). Intracranial implantation with subsequent 3D in vivo bioluminescent imaging of murine gliomas. J. Vis. Exp..

[23] Tsoli M., Chen Y., Gopalakrishnan A., Ung C., Khan A., Upton D., Ziegler D.S. (2025). Establishment of Orthotopic Patient-derived Xenograft Models for Brain Tumors using a Stereotaxic Device. J. Vis. Exp..

[24] Noffsinger B., Witter A., Sheybani N., Xiao A., Manigat L., Zhong Q., Taori S., Harris T., Bullock T., Price R. (2021). Technical choices significantly alter the adaptive immune response against immunocompetent murine gliomas in a model-dependent manner. J. Neurooncol..

[25] Yabo Y.A., Moreno-Sanchez P.M., Pires-Afonso Y., Kaoma T., Nosirov B., Scafidi A., Ermini L., Lipsa A., Oudin A., Kyriakis D. (2024). Glioblastoma-instructed microglia transition to heterogeneous phenotypic states with phagocytic and dendritic cell-like features in patient tumors and patient-derived orthotopic xenografts. Genome Med..

[26] Chenna S.S., Gajula S.N.R., Nalla L.V. (2025). Polyamine metabolism in cancer: Drivers of immune evasion, ferroptosis and therapy resistance. Expert Rev. Mol. Med..

[27] Wu J.Y., Zeng Y., You Y.Y., Chen Q.Y. (2025). Polyamine metabolism and anti-tumor immunity. Front. Immunol..

[28] Stockwell B.R., Friedmann Angeli J.P., Bayir H., Bush A.I., Conrad M., Dixon S.J., Fulda S., Gascón S., Hatzios S.K., Kagan V.E. (2017). Ferroptosis: A Regulated Cell Death Nexus Linking Metabolism, Redox Biology, and Disease. Cell.

[29] Kraft V.A.N., Bezjian C.T., Pfeiffer S., Ringelstetter L., Müller C., Zandkarimi F., Merl-Pham J., Bao X., Anastasov N., Kössl J. (2020). GTP Cyclohydrolase 1/Tetrahydrobiopterin Counteract Ferroptosis through Lipid Remodeling. ACS Cent. Sci..

[30] Wei Y., Xu Y., Sun Q., Hong Y., Liang S., Jiang H., Zhang X., Zhang S., Chen Q. (2024). Targeting ferroptosis opens new avenues in gliomas. Int. J. Biol. Sci..

[31] Liu T., Zhu C., Chen X., Guan G., Zou C., Shen S., Wu J., Wang Y., Lin Z., Chen L. (2022). Ferroptosis, as the most enriched programmed cell death process in glioma, induces immunosuppression and immunotherapy resistance. Neuro Oncol..

[32] Han L., Wang Z., Jiang Y., Zhou H., Zhou H. (2026). Ferroptosis and metabolic reprogramming in the immunosuppressive microenvironment of glioblastoma: Emerging mechanisms and novel strategies. Front. Immunol..

