# Supplementary material for: Spatial Association Between Polyamine Metabolism and Lipid Peroxidation in Different Pathological Regions of Glioblastoma
Source: Biomedicines. 2026 Sep 15;14(9):2067. doi: 10.3390/biomedicines14092067 (PMC13604745; doi:10.3390/biomedicines14092067)
Supplement: Supplementary file 1 [file biomedicines-14-02067-s001.zip › biomedicines-4545319-supplementary.pdf]

Supplementary information

Spatial association between polyamine metabolism and lipid peroxidation in different pathological regions of glioblastoma.

Zheng Qu 1,†, Kaijing Guo 2,†, Xin Xiang 3,†, Yuhan An 1, Ting Lei 3, Angsi Liu 4, Yulin Wang 1, Dongting Chen 1, Hong Chen 3, Ziqian Zhang 3, Jiangjia Qin 3, Jiuming He 2, Xueji Li 4, Fangjun Liu 3,* and Yan Li 1,*

1 Beijing Key Laboratory of New Drug Mechanisms and Pharmacological Evaluation Study, Department of Pharmacology, Institute of Materia Medica, Chinese Academy of Medical Sciences & Peking Union Medical College, Beijing 100050, China

2 State Key Laboratory of Bioactive Substance and Function of Natural Medicines, Institute of Materia Medica, Chinese Academy of Medical Sciences & Peking Union Medical College, Beijing 100050, China; hejiuming@imm.ac.cn (J.H.)

3 Department of Neurosurgery, Sanbo Brain Hospital, Capital Medical University, Beijing 100018, China

4 Department of Neurosurgery, National Cancer Center/National Clinical Research Center for Cancer/Cancer Hospital, Chinese Academy of Medical Sciences & Peking Union Medical College, Beijing 100021, China; li-x-j@163.com (X.L.)

* Correspondence: liufangjun@mail.ccmu.edu.cn (F.L.); liyanxiao@imm.ac.cn (Y.L.)

† These authors contributed equally to this work.

Table S1: Patient information

patient ID	sex	age	diagnosis	presentation	test item	Corresponding figures	CNS WHO grade	IDH status	ATRX status	H3K27M status	Diagnostic note
1	M	45	GBM	primary	spatial metabolomics, FCM	Fig 1, 2B, 2C, 4	4	wild type	wild type	wild type	
2	M	69	GBM	primary	spatial metabolomics, FCM, IHC	Fig 1, 2B, 2C, 3, 4	4	wild type	wild type	wild type	
3	M	58	GBM	primary	spatial metabolomics, FCM, IHC	Fig 1, 2B, 2C, 2D, 2E, 3A-3C, 4	4	wild type	wild type	wild type	
4	F	66	GBM	primary	spatial metabolomics, IHC	Fig 1, 2B, 2C, 3A-3C	4	wild type	wild type	wild type	
5	M	68	GBM	primary	spatial metabolomics, IHC	Fig 1, 2B, 2C, 3A-3C	4	wild type	wild type	wild type	
6	M	56	GBM	recurrent	spatial metabolomics, IHC	Fig 1, 2B, 2C, 2F, 2G, 3A-3C	4	wild type	wild type	wild type	
7	M	46	GBM	recurrent	spatial metabolomics, FCM, IHC	Fig 1, 2B, 2C, 3A-3C, 4	4	/	/	/	
8	M	54	GBM	recurrent	spatial metabolomics, FCM, IHC	Fig 1, 2B, 2C, 3, 4	4	wild type	mutant type	mutant type	Exploratory case
9	M	61	GBM	recurrent	spatial metabolomic, FCM, IHC	Fig 1, 2B, 2C, 3A-3C, 4	3	/	/	/	Exploratory case
10	M	47	GBM	primary	FCM	Fig 4	4	mutant type	mutant type	wild type	Exploratory case
11	M	30	GBM	primary	FCM	Fig 4	3-4	/	/	/	
12	F	68	GBM	primary	IHC, IF	Fig 3A-3C, 5	4	wild type	wild type	wild type	
13	F	68	GBM	recurrent	IHC, FCM, IF	Fig 3A-3C, 4, 5	4	wild type	wild type	wild type	
14	M	58	GBM	recurrent	FCM	Fig 4	2	/	/	/	Exploratory case
15	F	74	GBM	primary	FCM	Fig 4	4	wild type	wild type	wild type	
16	F	58	GBM	primary	FCM	Fig 4	3	/	/	/	Exploratory case
17	M	53	GBM	primary	FCM	Fig 4	4	wild type	wild type	wild type	

M stands for male and F stands for female. The data related sex were self-reported by study participants. Most primary and recurrent GBM specimens in this cohort are obtained from different patients; paired longitudinal primary-recurrent samples from the same individual are limited. Full prior-treatment history is not available for all archived cases. Differences observed between primary and recurrent groups cannot be exclusively interpreted as consequences of tumor recurrence, and may be confounded by inter-patient heterogeneity, molecular subtypes, tumor location, sample processing, and prior therapeutic interventions. Cases marked as exploratory do not satisfy strict WHO diagnostic criteria for glioblastoma. Patient 12 and Patient 13 are primary-recurrent longitudinal specimens originating from one single patient. Two distinct biobank sample IDs were assigned because the two tumour tissues were obtained from two separate surgical operations and submitted independently to the sample biobank.

Table S2 Immune cell counts and proportions in patients with primary and recurrent GBM

Patient ID	Type	Location	CD3+		CD4+		PD-1（CD4+）		CD8+		PD-1（CD8+）	
			count	parent（%）	count	parent（%）	count	parent（%）	count	parent（%）	count	parent（%）
10	primary	P	62↓	0.11	5↓	8.06	1↓	20	2↓	3.23	2↓	100
		T	215	4.29	24↓	11.16	21↓	87.5	12↓	5.58	12↓	100
11	primary	P	241	0.18	0↓	0	0↓	0	22	9.13	22↓	100
		T	4376	3.7	2594	54.77	2417	93.18	264	5.57	235	89.02
1	primary	P	1170	0.84	1060	90.6	418	39.43	0↓	0	0↓	0
		T	14245	48.07	8751	61.43	7463	85.28	0↓	0	0↓	0
2	primary	P	511	0.93	498	97.46	446	89.56	0↓	0	0↓	0
		T	3155	49.27	384	12.17	255	66.41	17↓	0.54	7↓	41.18
3	primary	P	1779	0.87	1571	88.31	1567	99.75	0↓	0	0↓	0
		T	1615	1.22	1480	91.64	1439	97.23	0↓	0	0↓	0
15	primary	P	169	0.05	55↓	32.54	39↓	70.91	81↓	47.93	67↓	82.72
		T	135	0.09	54↓	40	34↓	62.96	50↓	37.04	42↓	84
16	primary	P	90↓	0.05	21↓	23.33	6↓	28.57	37↓	41.11	26↓	70.27
		T	792	2.42	310	39.14	160	51.61	276	34.85	97↓	35.14
17	primary	P	18↓	0.01	1↓	5.56	0↓	0	2↓	11.11	1↓	50
		T	16645	12.86	9672	56.11	8565	88.55	5379	32.32	4883	90.78
7	recurrent	P	212	0.1	206	97.17	47↓	22.82	0↓	0	0↓	0
		T	11134	40.62	8696	78.1	4496	40.19	0↓	0	0↓	0
8	recurrent	P	399	0.31	375	93.98	112	29.87	0↓	0	0↓	0
		T	972	0.52	354	36.42	289	81.64	541	55.66	489	90.39
9	recurrent	T	2081	0.51	1753	84.24	1691	96.46	0↓	0	0↓	0
13	recurrent	P	25↓	0.02	2↓	8	0↓	0	1↓	4	0↓	0
		T	136	0.04	14↓	10.29	14↓	100	40↓	29.41	31↓	77.5
14	recurrent	P	8944	2.99	3964	44.32	3284	82.85	4419	49.41	3622	81.96
		T	5798	1.83	2734	47.15	2144	78.42	2600	44.84	2282	87.77

↓: Value calculated from fewer than 100 events within the gated target immune subset (denominator for percentage calculation); these measurements are regarded as unreliable and excluded from statistical analysis.

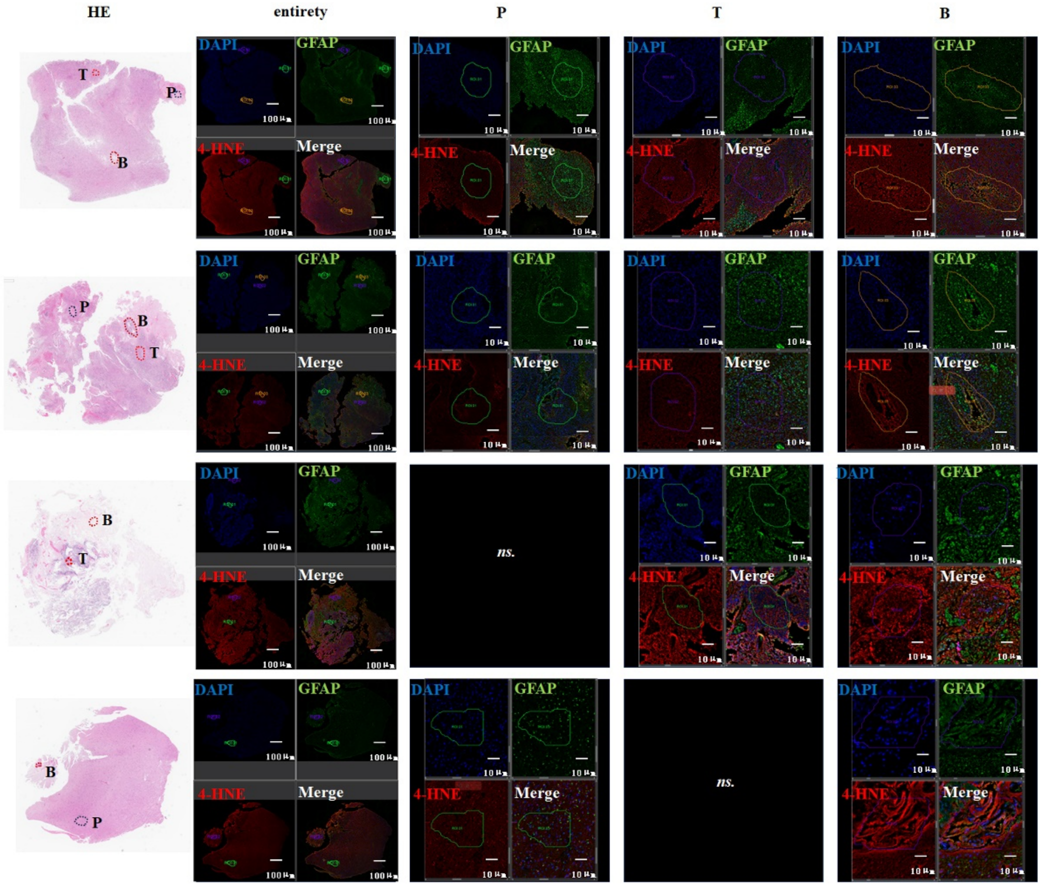

Figure S1: The regional expression of the 4-HNE in the primary GBM patients (patient ID: 2, 3, 4, 5, these specimens are included in the quantitative summary shown in Figure 3C.). Patient 4 does not have the typical P region and patient 5 does not have the typical T region. For statistical comparison, only valid pathologically confirmed regions from each individual were included; missing or atypical regions were not imputed, interpolated, or artificially supplemented, and were excluded from corresponding regional pairwise analyses. Red represents 4-HNE, green represents GFAP, and blue represents DAPI.

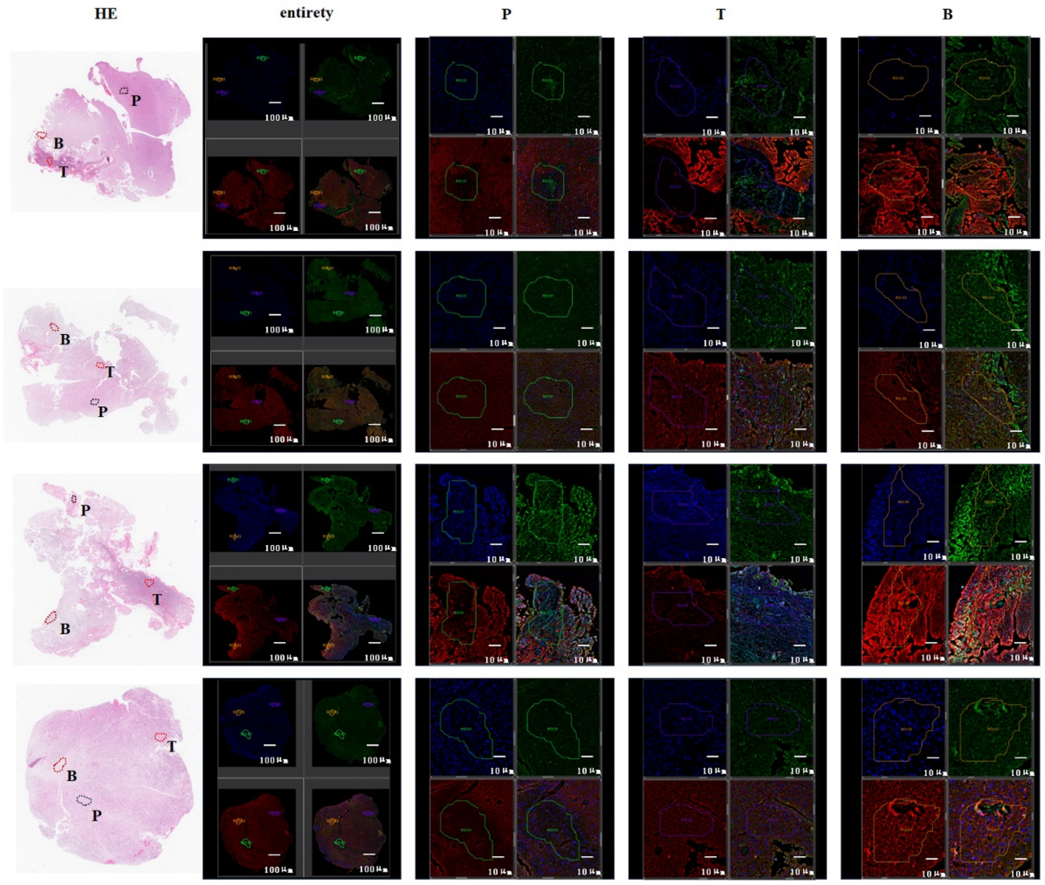

Figure S2: The regional expression of the 4-HNE in the recurrent GBM patients (patient ID: 6, 7, 8, 13, these specimens are included in the quantitative summary shown in Figure 3C.) Red represents 4-HNE, green represents GFAP, and blue represents DAPI.

Supplementary Table S3 on pathway enrichment (Fig1D T vs P)

	total	expected	hits	Raw p	Holm p	FDR
Tyrosine metabolism	42	12.7	32	6.01e-10	4.87e-08	4.87e-08
Arachidonic acid metabolism	44	13.3	30	1.52e-07	1.22e-05	6.16e-06
Galactose metabolism	26	7.86	19	6.86e-06	0.000542	0.000185
Arginine and proline metabolism	35	10.6	23	1.23e-05	0.000962	0.00025
D-Amino acid metabolism	14	4.23	12	2.51e-05	0.00194	0.000407
Pentose phosphate pathway	22	6.65	16	4.28e-05	0.00326	0.000578
Valine, leucine and isoleucine biosynthesis	8	2.42	8	6.66e-05	0.00499	0.000674
Phenylalanine metabolism	8	2.42	8	6.66e-05	0.00499	0.000674
Arginine biosynthesis	14	4.23	11	0.000248	0.0181	0.00216
Histidine metabolism	16	4.83	12	0.000266	0.0192	0.00216
Glycine, serine and threonine metabolism	32	9.67	19	0.000516	0.0367	0.0038
Amino sugar and nucleotide sugar metabolism	31	9.37	18	0.00105	0.0737	0.00711
Starch and sucrose metabolism	18	5.44	12	0.00144	0.0994	0.00897
Steroid hormone biosynthesis	86	26	39	0.00165	0.112	0.00952
beta-Alanine metabolism	21	6.35	13	0.00245	0.164	0.0132
Pentose and glucuronate interconversions	19	5.74	12	0.00285	0.188	0.0144
Pantothenate and CoA biosynthesis	20	6.04	12	0.0052	0.338	0.0248
Alanine, aspartate and glutamate metabolism	28	8.46	15	0.00781	0.5	0.0351
Phenylalanine, tyrosine and tryptophan biosynthesis	4	1.21	4	0.00826	0.52	0.0352
Fructose and mannose metabolism	21	6.35	12	0.00887	0.55	0.0359
Caffeine metabolism	10	3.02	7	0.0108	0.66	0.0417
Linoleic acid metabolism	5	1.51	4	0.0314	1	0.113
Glycolysis / Gluconeogenesis	24	7.25	12	0.0321	1	0.113

Nicotinate and nicotinamide metabolism	15	4.53	8	0.0512	1	0.173
Ascorbate and aldarate metabolism	11	3.32	6	0.08	1	0.259
Neomycin, kanamycin and gentamicin biosynthesis	2	0.604	2	0.0912	1	0.284
Glutathione metabolism	28	8.46	12	0.105	1	0.316
Citrate cycle (TCA cycle)	20	6.04	9	0.116	1	0.33
Tryptophan metabolism	40	12.1	16	0.118	1	0.33
One carbon pool by folate	26	7.86	11	0.129	1	0.345
Butanoate metabolism	15	4.53	7	0.134	1	0.345
Glyoxylate and dicarboxylate metabolism	32	9.67	13	0.136	1	0.345
Cysteine and methionine metabolism	33	9.97	13	0.166	1	0.401
Pyrimidine metabolism	39	11.8	15	0.168	1	0.401
Taurine and hypotaurine metabolism	8	2.42	4	0.198	1	0.457
Vitamin B6 metabolism	9	2.72	4	0.275	1	0.618
Glycerolipid metabolism	16	4.83	6	0.347	1	0.755
Purine metabolism	70	21.2	23	0.355	1	0.755
Inositol phosphate metabolism	32	9.67	11	0.365	1	0.755
Lysine degradation	29	8.76	10	0.373	1	0.755
Retinol metabolism	17	5.14	6	0.411	1	0.811
Drug metabolism - cytochrome P450	55	16.6	17	0.507	1	0.971
Pyruvate metabolism	22	6.65	7	0.515	1	0.971
Biosynthesis of unsaturated fatty acids	36	10.9	11	0.546	1	1
Valine, leucine and isoleucine degradation	40	12.1	12	0.573	1	1
Glycosylphosphatidylinositol (GPI)-anchor biosynthesis	6	1.81	2	0.585	1	1
Nitrogen metabolism	6	1.81	2	0.585	1	1
Biosynthesis of various nucleotide sugars	24	7.25	7	0.622	1	1

Glycosaminoglycan biosynthesis - chondroitin sulfate / dermatan sulfate	3	0.907	1	0.66	1	1
Thiamine metabolism	7	2.12	2	0.676	1	1
Terpenoid backbone biosynthesis	18	5.44	5	0.676	1	1
Propanoate metabolism	22	6.65	6	0.696	1	1
Folate biosynthesis	26	7.86	7	0.714	1	1
Mannose type O-glycan biosynthesis	4	1.21	1	0.763	1	1
Sphingolipid metabolism	33	9.97	8	0.828	1	1
N-Glycan biosynthesis	10	3.02	2	0.855	1	1
Biotin metabolism	10	3.02	2	0.855	1	1
Primary bile acid biosynthesis	46	13.9	11	0.867	1	1
Phosphonate and phosphinate metabolism	6	1.81	1	0.885	1	1
Glycerophospholipid metabolism	36	10.9	8	0.896	1	1
Ubiquinone and other terpenoid-quinone biosynthesis	20	6.04	4	0.898	1	1
Lipoic acid metabolism	28	8.46	5	0.956	1	1
Ether lipid metabolism	20	6.04	3	0.967	1	1
Porphyrin metabolism	31	9.37	5	0.978	1	1
Steroid biosynthesis	41	12.4	7	0.983	1	1
alpha-Linolenic acid metabolism	13	3.93	1	0.991	1	1
Selenocompound metabolism	20	6.04	2	0.993	1	1
Drug metabolism - other enzymes	39	11.8	3	1	1	1
Fatty acid biosynthesis	47	14.2	4	1	1	1
Fatty acid degradation	39	11.8	2	1	1	1
Fatty acid elongation	38	11.5	1	1	1	1
Metabolism of xenobiotics by cytochrome P450	68	20.5	5	1	1	1

Supplementary Table S4 on pathway enrichment (Fig1D T vs B)

	total	expected	hits	Raw p	Holm p	FDR
Tyrosine metabolism	42	13.4	31	1.76e-08	1.42e-06	1.42e-06
Arachidonic acid metabolism	44	14	31	1.06e-07	8.49e-06	4.3e-06
Arginine and proline metabolism	35	11.1	24	6.88e-06	0.000544	0.000186
Pentose phosphate pathway	22	7	17	1.33e-05	0.00104	0.000236
Steroid hormone biosynthesis	86	27.3	46	1.45e-05	0.00112	0.000236
D-Amino acid metabolism	14	4.45	12	4.48e-05	0.00341	0.000605
Valine, leucine and isoleucine biosynthesis	8	2.54	8	1e-04	0.00753	0.00102
Phenylalanine metabolism	8	2.54	8	1e-04	0.00753	0.00102
Galactose metabolism	26	8.27	17	0.000399	0.0291	0.00333
Arginine biosynthesis	14	4.45	11	0.000411	0.0296	0.00333
Histidine metabolism	16	5.09	12	0.000456	0.0323	0.00335
Amino sugar and nucleotide sugar metabolism	31	9.86	19	0.000605	0.0424	0.00408
Pentose and glucuronate interconversions	19	6.04	13	0.00107	0.0737	0.00666
Glycine, serine and threonine metabolism	32	10.2	18	0.00332	0.226	0.0192
Pantothenate and CoA biosynthesis	20	6.36	12	0.00824	0.552	0.0445
Starch and sucrose metabolism	18	5.72	11	0.00946	0.625	0.0479
Phenylalanine, tyrosine and tryptophan biosynthesis	4	1.27	4	0.0101	0.659	0.0483
Fructose and mannose metabolism	21	6.68	12	0.0138	0.882	0.062
Caffeine metabolism	10	3.18	7	0.0147	0.923	0.0625
Alanine, aspartate and glutamate metabolism	28	8.9	14	0.0329	1	0.133
Linoleic acid metabolism	5	1.59	4	0.0379	1	0.144
beta-Alanine metabolism	21	6.68	11	0.0391	1	0.144
Butanoate metabolism	15	4.77	8	0.0678	1	0.239

Taurine and hypotaurine metabolism	8	2.54	5	0.0726	1	0.245
One carbon pool by folate	26	8.27	12	0.0874	1	0.283
Ascorbate and aldarate metabolism	11	3.5	6	0.0995	1	0.303
Neomycin, kanamycin and gentamicin biosynthesis	2	0.636	2	0.101	1	0.303
Cysteine and methionine metabolism	33	10.5	14	0.129	1	0.373
Glutathione metabolism	28	8.9	12	0.144	1	0.403
Citrate cycle (TCA cycle)	20	6.36	9	0.151	1	0.406
Tryptophan metabolism	40	12.7	16	0.169	1	0.441
Primary bile acid biosynthesis	46	14.6	18	0.177	1	0.448
Glycolysis / Gluconeogenesis	24	7.63	10	0.202	1	0.496
Pyrimidine metabolism	39	12.4	15	0.23	1	0.547
Glyoxylate and dicarboxylate metabolism	32	10.2	12	0.3	1	0.695
Vitamin B6 metabolism	9	2.86	4	0.312	1	0.702
Nicotinate and nicotinamide metabolism	15	4.77	6	0.332	1	0.728
Purine metabolism	70	22.3	24	0.367	1	0.783
Pyruvate metabolism	22	7	8	0.398	1	0.811
Glycerolipid metabolism	16	5.09	6	0.4	1	0.811
Lysine degradation	29	9.22	10	0.446	1	0.881
Retinol metabolism	17	5.41	6	0.468	1	0.902
Steroid biosynthesis	41	13	13	0.565	1	1
Inositol phosphate metabolism	32	10.2	10	0.594	1	1
Drug metabolism - cytochrome P450	55	17.5	17	0.609	1	1
Glycosylphosphatidylinositol (GPI)-anchor biosynthesis	6	1.91	2	0.618	1	1
Nitrogen metabolism	6	1.91	2	0.618	1	1
Folate biosynthesis	26	8.27	8	0.619	1	1

Biosynthesis of unsaturated fatty acids	36	11.4	11	0.627	1	1
Valine, leucine and isoleucine degradation	40	12.7	12	0.656	1	1
Glycosaminoglycan biosynthesis - chondroitin sulfate / dermatan sulfate	3	0.954	1	0.683	1	1
Biosynthesis of various nucleotide sugars	24	7.63	7	0.684	1	1
Thiamine metabolism	7	2.23	2	0.708	1	1
Terpenoid backbone biosynthesis	18	5.72	5	0.726	1	1
Propanoate metabolism	22	7	6	0.75	1	1
Mannose type O-glycan biosynthesis	4	1.27	1	0.784	1	1
Ubiquinone and other terpenoid-quinone biosynthesis	20	6.36	5	0.814	1	1
Sphingolipid metabolism	33	10.5	8	0.873	1	1
N-Glycan biosynthesis	10	3.18	2	0.878	1	1
Phosphonate and phosphinate metabolism	6	1.91	1	0.9	1	1
Glycerophospholipid metabolism	36	11.4	8	0.928	1	1
alpha-Linolenic acid metabolism	13	4.13	2	0.952	1	1
Ether lipid metabolism	20	6.36	3	0.976	1	1
Biotin metabolism	10	3.18	1	0.979	1	1
Lipoic acid metabolism	28	8.9	4	0.991	1	1
Selenocompound metabolism	20	6.36	2	0.995	1	1
Porphyrin metabolism	31	9.86	4	0.996	1	1
Drug metabolism - other enzymes	39	12.4	5	0.999	1	1
Fatty acid biosynthesis	47	14.9	4	1	1	1
Fatty acid degradation	39	12.4	2	1	1	1
Fatty acid elongation	38	12.1	1	1	1	1
Metabolism of xenobiotics by cytochrome P450	68	21.6	4	1	1	1

Supplementary Table S5 on pathway enrichment (Fig 6B)

	total	expected	hits	Raw p	Holm p	FDR
Arginine and proline metabolism	35	0.507	10	5.65e-12	4.58e-10	4.58e-10
Glutathione metabolism	28	0.406	7	4.6e-08	3.68e-06	1.86e-06
Arginine biosynthesis	14	0.203	5	7.24e-07	5.72e-05	1.95e-05
Lysine degradation	29	0.42	4	0.000619	0.0483	0.0125
D-Amino acid metabolism	14	0.203	3	0.000867	0.0667	0.014
beta-Alanine metabolism	21	0.304	3	0.00296	0.225	0.04
Alanine, aspartate and glutamate metabolism	28	0.406	2	0.0603	1	0.698
Linoleic acid metabolism	5	0.0724	1	0.0704	1	0.713
Biotin metabolism	10	0.145	1	0.136	1	1
Nicotinate and nicotinamide metabolism	15	0.217	1	0.197	1	1
Histidine metabolism	16	0.232	1	0.209	1	1
Citrate cycle (TCA cycle)	20	0.29	1	0.254	1	1
Pantothenate and CoA biosynthesis	20	0.29	1	0.254	1	1
Pyruvate metabolism	22	0.319	1	0.276	1	1
One carbon pool by folate	26	0.377	1	0.318	1	1
Lipoic acid metabolism	28	0.406	1	0.338	1	1
Porphyrin metabolism	31	0.449	1	0.367	1	1
Glycine, serine and threonine metabolism	32	0.463	1	0.376	1	1
Glyoxylate and dicarboxylate metabolism	32	0.463	1	0.376	1	1
Biosynthesis of unsaturated fatty acids	36	0.521	1	0.412	1	1
Tyrosine metabolism	42	0.608	1	0.463	1	1
Primary bile acid biosynthesis	46	0.666	1	0.494	1	1
